# UBASH3B-mediated MRPL12 Y60 dephosphorylation inhibits LUAD development by driving mitochondrial metabolism reprogramming

**DOI:** 10.1186/s13046-024-03181-x

**Published:** 2024-09-30

**Authors:** Xingzhao Ji, Tianyi Zhang, Jian Sun, Xiaojia Song, Guoyuan Ma, Li Xu, Xueru Cao, yongjian jing, Fuyuan Xue, Weiying Zhang, Shengnan Sun, Qiang Wan, Yi Liu

**Affiliations:** 1 Department of Pulmonary and Critical Care Medicine, Shandong Provincial Hospital Affiliated to Shandong First Medical University, Jinan, Shandong 250021 China; 2 Shandong Provincial Key Medical and Health Laboratory of Cell Metabolism, Central Hospital Affiliated to Shandong First Medical University, Jinan, Shandong 250021 China; 3 Medical Science and Technology Innovation Center, Shandong First Medical University & Shandong Academy of Medical Sciences, Jinan, Shandong China; 4 Department of Thoracic Surgery Department, Shandong Provincial Hospital Affiliated to Shandong First Medical University, Jinan, Shandong 250021 China; 5 Department of Pulmonary and Critical Care Medicine, Heze Municipal Hospital, Heze, Shandong 274000 China; 6 Department of Pulmonary and Critical Care Medicine, the First People’s Hospital of Pingyuan, Dezhou, Shandong 253000 China

**Keywords:** MRPL12, Metabolic reprogramming, Oxidative phosphorylation, LUAD, UBASH3B

## Abstract

**Background:**

Metabolic reprogramming plays a pivotal role in tumorigenesis and development of lung adenocarcinoma (LUAD). However, the precise mechanisms and potential targets for metabolic reprogramming in LUAD remain elusive. Our prior investigations revealed that the mitochondrial ribosomal protein MRPL12, identified as a novel mitochondrial transcriptional regulatory gene, exerts a critical influence on mitochondrial metabolism. Despite this, the role and regulatory mechanisms underlying MRPL12’s transcriptional activity in cancers remain unexplored.

**Methods:**

Human LUAD tissues, *Tp53*^*fl/fl*^;*Kras*^*G12D*^-driven LUAD mouse models, LUAD patient-derived organoids (PDO), and LUAD cell lines were used to explored the expression and function of MRPL12. The posttranslational modification of MRPL12 was analyzed by mass spectrometry, and the oncogenic role of key phosphorylation sites of MRPL12 in LUAD development was verified in vivo and in vitro.

**Results:**

MRPL12 was upregulated in human LUAD tissues, *Tp53*^*fl/fl*^;*Kras*^*G12D*^-driven LUAD tissues in mice, LUAD PDO, and LUAD cell lines, correlating with poor patient survival. Overexpression of MRPL12 significantly promoted LUAD tumorigenesis, metastasis, and PDO formation, while MRPL12 knockdown elicited the opposite phenotype. Additionally, MRPL12 deletion in a *Tp53*^*fl/fl*^;*Kras*^*G12D*^-driven mouse LUAD model conferred a notable survival advantage, delaying tumor onset and reducing malignant progression. Mechanistically, we discovered that MRPL12 promotes tumor progression by upregulating mitochondrial oxidative phosphorylation. Furthermore, we identified UBASH3B as a specific binder of MRPL12, dephosphorylating tyrosine 60 in MRPL12 (MRPL12 Y60) and inhibiting its oncogenic functions. The decrease in MRPL12 Y60 phosphorylation impeded the binding of MRPL12 to POLRMT, downregulating mitochondrial metabolism in LUAD cells. In-depth in vivo, in vitro, and organoid models validated the inhibitory effect of MRPL12 Y60 mutation on LUAD.

**Conclusion:**

This study establishes MRPL12 as a novel oncogene in LUAD, contributing to LUAD pathogenesis by orchestrating mitochondrial metabolism reprogramming towards oxidative phosphorylation (OXPHOS). Furthermore, it confirms Y60 as a specific phosphorylation modification site regulating MRPL12’s oncogenic functions, offering insights for the development of LUAD-specific targeted drugs and clinical interventions.

**Graphical Abstract:**

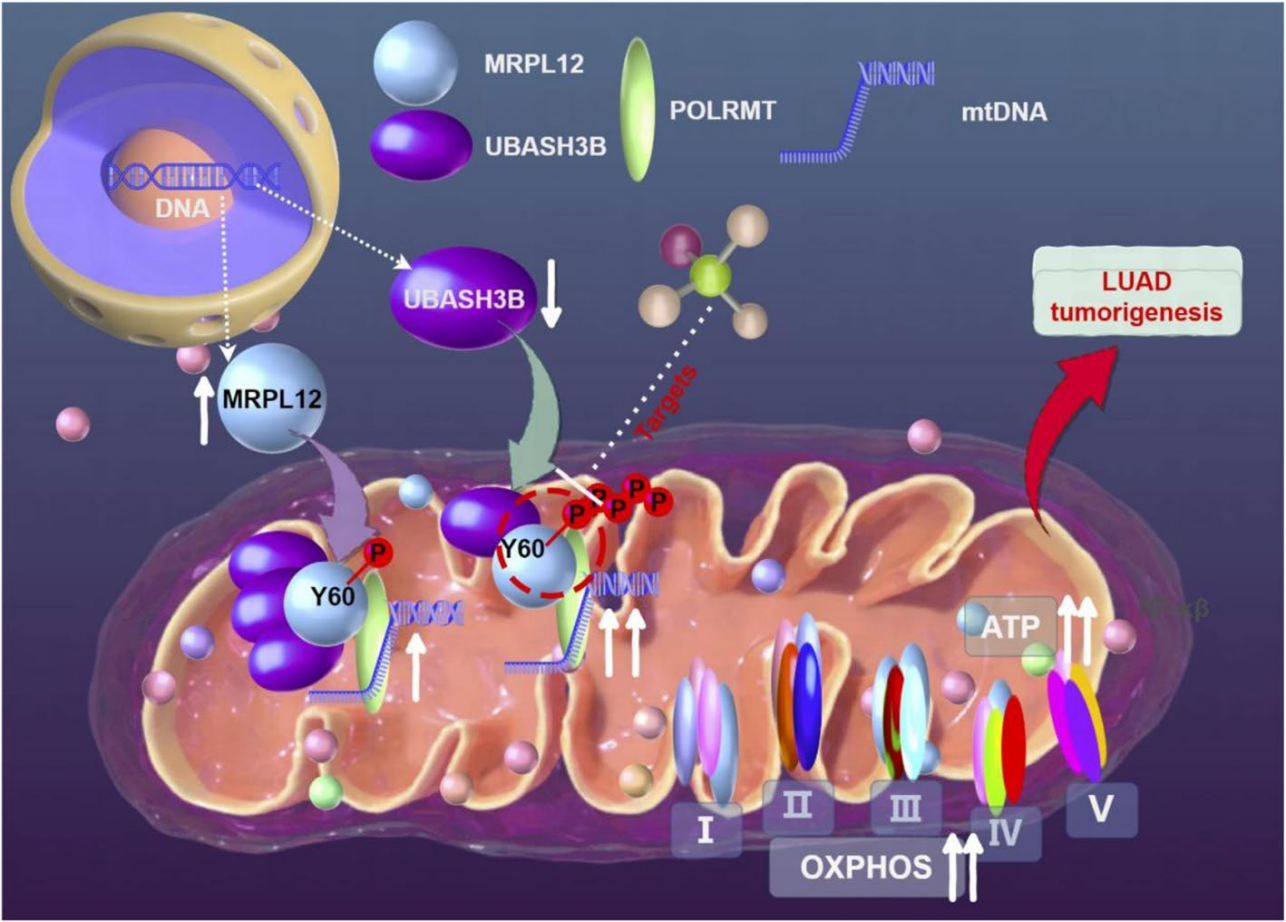

**Supplementary Information:**

The online version contains supplementary material available at 10.1186/s13046-024-03181-x.

## Introduction

Lung cancer is the first leading cause of cancer-related death worldwide and non-small cell lung cancer (NSCLC) is the major histopathology subtype of lung cancer [1]. LUAD, characterized by substantial morbidity and mortality, constitutes the predominant subtype within NSCLC [2, 3]. Despite the utilization of various treatment modalities such as surgery, chemotherapy, immunotherapy, and molecular targeted therapy, the prognosis for LUAD patients remains unfavorable [4, 5]. Thus, LUAD diagnosis and treatment remain major challenges and identify potential new therapeutic targets is urgent, which rely on a better understanding of the tumorigenesis mechanisms of LUAD.

Metabolic reprogramming, a key feature acquired during malignant transformation, stands as a hallmark of cancer [6]. For decades, the study of metabolic reprogramming primarily revolved around the “Warburg effect,” widely recognized as a metabolic signature in cancers. This effect manifests as abnormal metabolism, characterized by heightened glycolysis instead of OXPHOS, irrespective of abundant oxygen availability [7]. Recently, there has been a growing emphasis on the dysregulation of mitochondrial metabolism, particularly OXPHOS, in the tumorigenesis of LUAD [8–10]. Research by Han et al. demonstrated that LUAD cells exhibit significantly elevated OXPHOS characteristics, suggesting a potential reliance on OXPHOS for their energy demands [11]. Furthermore, RNA-Seq and proteome analyses of LUAD tissue indicated a substantial upregulation of OXPHOS [12, 13]. Crucially, it has been established that OXPHOS could serve as a viable target in cancer therapy [14]. In summary, the role of mitochondrial OXPHOS in LUAD tumorigenesis is pivotal. However, the specific regulatory mechanisms governing mitochondrial OXPHOS in LUAD remain incompletely understood, and potential intervention targets remain unclear.

MRPL12 (mitochondrial ribosomal protein L7/L12) is the first mitochondrial ribosomal protein identified in mammals [15]. Recent insights highlight MRPL12’s multifaceted role; it not only partakes in the translation of mitochondrial proteins but also activates mtDNA transcription by directly engaging with mitochondrial RNA polymerase POLRMT. This interaction initiates mitochondrial biogenesis, orchestrating the regulation of mitochondrial OXPHOS and cellular energy provisioning [16, 17]. Significantly, our prior investigations have linked MRPL12-mediated regulation of mitochondrial biosynthesis, OXPHOS, and metabolism to the pathogenesis of diverse metabolic disorders, including ischemic and hypoxic conditions, as well as diabetes [18, 19]. Furthermore, our scrutiny has delved into the transcriptional regulatory and post-translational modification mechanisms of MRPL12 in diabetic kidney disease [20]. Collectively, these studies underscore MRPL12’s involvement in the development of metabolically related diseases through the governance of mitochondrial metabolism and OXPHOS. However, the specific association of MRPL12 with mitochondrial metabolism, particularly OXPHOS, in LUAD, and the molecular underpinnings in the tumorigenesis of LUAD, remain elusive.

Accumulating evidence underscores the pivotal role of posttranslational modifications (PTM) in governing the physiological functions of oncoproteins. These modifications, altering protein activity, stability, and interactions, have been implicated in the tumorigenesis of various cancers [21–23]. Notably, the targeted modulation of PTMs holds promising clinical significance across diverse tumor interventions [24]. For instance, small molecule inhibitors targeting the phosphorylation of specific proteins can selectively modulate their physiological functions, thereby impeding tumor growth or inducing apoptosis in cancer cells [25, 26]. Our previous investigations revealed that CUL3-mediated ubiquitination modification of MRPL12 influences MRPL12’s protein stability, subsequently leading to dysregulated mitochondrial biosynthesis in renal tubular epithelial cells, contributing to the onset and progression of diabetic nephropathy [20]. However, it remains unclear whether abnormal PTMs of MRPL12 exist, capable of influencing mitochondrial OXPHOS in LUAD, and whether these MRPL12 PTMs could serve as potential intervention targets for LUAD therapy.

In the current study, we elucidated the pivotal role of MRPL12 in metabolic reprogramming within LUAD by using PDO, LSL-Kras^G12D^; LSL-p53^+/+^ LUAD mouse model, as well as LUAD tissues and cells. Our exploration further revealed the interaction between MRPL12 and Ubiquitin Associated and SH3 Domain Containing B (UBASH3B) tyrosine phosphorylase. This interaction modulates mitochondrial biosynthesis and metabolism by dephosphorylating MRPL12 at Y60, influencing the tumorigenesis of LUAD. These findings underscore the significance of MRPL12, a recently identified mitochondrial transcriptional regulator, in driving LUAD tumorigenesis through orchestrating metabolic reprogramming towards OXPHOS. Additionally, the phosphorylation of MRPL12 Y60 emerges as a potential therapeutic target for LUAD.

## Results

### Lung-specific ablation of MRPL12 suppresses lung tumorigenesis in Tp53^−/−^;Kras^G12D/+^ mice

**Fig. 1:**
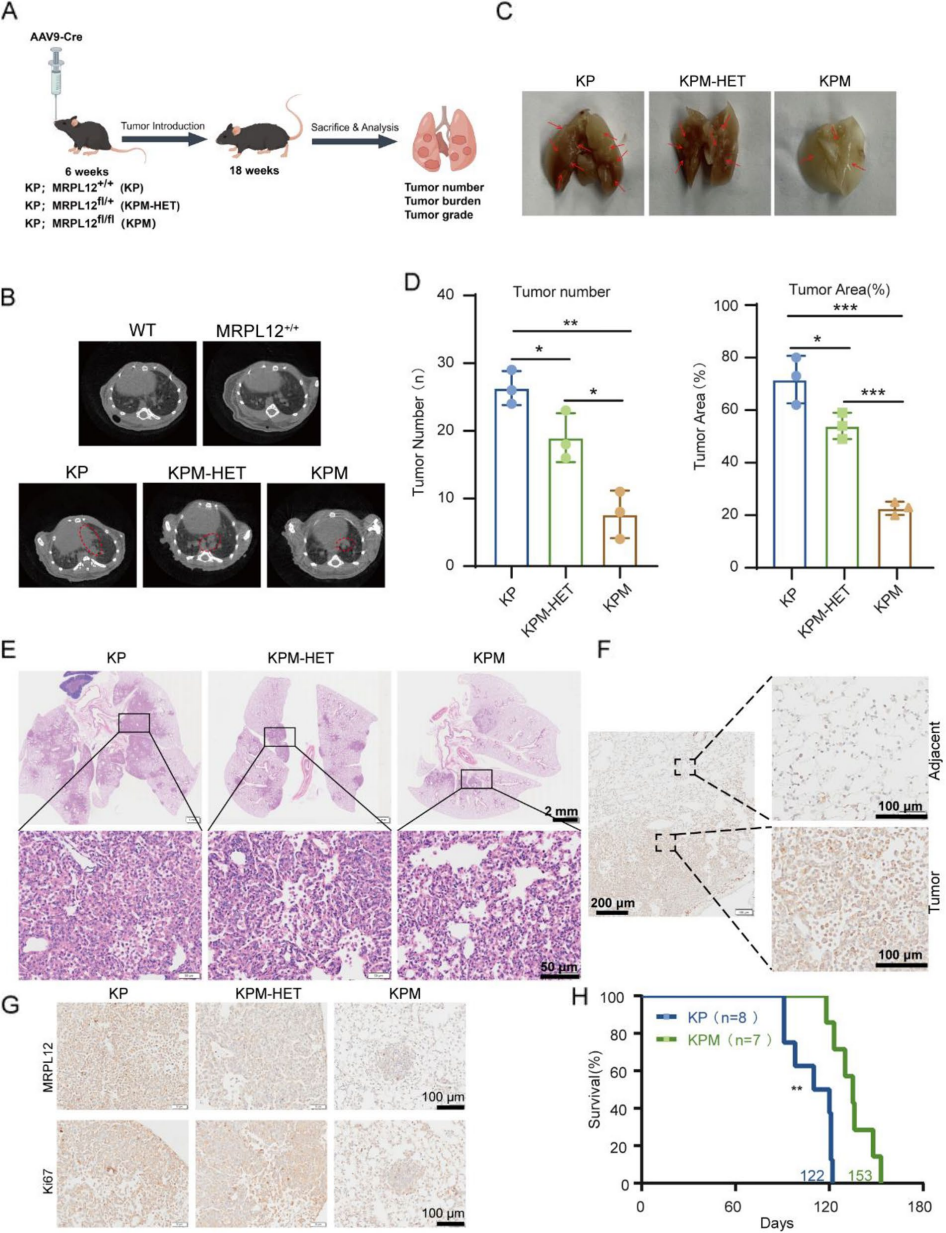
Lung-specific ablation of MRPL12 suppresses lung tumorigenesis in Tp53^−/−^;Kras^G12D/+^ mice. **A** Schematic depiction illustrating the induction of lung tumors in a genetically engineered mouse model. **B** Representative micro-CT images displaying lung morphology in of *Tp53*^*fl/fl*^*;Kras*^*G12D*^ (KP), *Tp53*^*fl/fl*^*;Kras*^*G12D*^*;MRPL12*^*fl/*+^(KPM-HET) and *Tp53*^*fl/fl*^*;Kras*^*G12D*^*;MRPL12*.^*fl/fl*^(KPM) mice. **C** Photomicrographs presenting lung tissues of KP, KPM-HET, and KPM mice following an 18-week inhalation of Adeno-Cre. Tumor lesions are demarcated by arrows. **D** Quantification of tumor nodules and their respective area (%) in lung tissues from KP, KPM-HET, and KPM mice. **E** HE-stained lung tissues obtained from KP, KPM-HET, and KPM mice. **F** Representative Immunohistochemical (IHC) staining of MRPL12 in lung sections from KP mice. **G** IHC staining of MRPL12 and Ki67 in lung sections from KP, KPM-HET, and KPM mice. **H** Survival rates of the KP and KPM mice, *n* = 8 and 7, respectively.*, *p* < 0.05; **, *p* < 0.01; ***, *p* < 0.001

KRAS is the predominant driver mutation in LUAD, and the deficiency of the Tp53 gene typically collaborates with the oncogenic activity of Kras, inducing LUAD [27, 28]. *Tp53*^−/−^;*Kras*^G12D/+^ mouse model is a well-established and widely used model of spontaneous LUAD, providing a robust platform for studying the disease’s progression [29–31]. By using genetic engineering techniques, researchers can induce mutations in the lungs of these mice to investigate the role of specific target genes in LUAD development and explore potential therapeutic strategies. To assess the impact of MRPL12 in LUAD, we bred *MRPL12*^*fl/fl*^ with *Tp53*^*fl/fl*^*;LSL-Kras*^*G12D*^ mice, generating *Tp53*^*fl/fl*^*;Kras*^*G12D*^ (KP), *Tp53*^*fl/fl*^*;Kras*^*G12D*^*;MRPL12*^*fl/*+^ (KPM-HET), and *Tp53*^*fl/fl*^*;Kras*^*G12D*^*;MRPL12*^*fl/fl*^ (KPM) mice. Adenoviral delivery of Cre recombinase (Ad-Cre) via intratracheal instillation with AAV9-CMV-Cre achieved Kras^G12D^ mutant activation and simultaneous deletion of Tp53 and MRPL12 (Fig. 1A). The progression of KP-driven LUAD was tracked through noninvasive microcomputed tomography (micro-CT) and hematoxylin–eosin (HE) staining. Eighteen weeks post Ad-Cre administration, tumor burden was assessed. Micro-CT illustrated substantial tumor burden in Ad-Cre-treated KP mice (Fig. 1B), visible as nodules upon gross lung inspection (Fig. 1C and D). Conversely, both heterozygous and homozygous MRPL12 knockout mice exhibited significantly reduced tumor burden (Fig. 1B, C, and D). In line with micro-CT, HE staining revealed decreased tumor burden, smaller nodule size, and fewer nodules in both heterozygous and homozygous MRPL12 knockout mice (Fig. 1E). KP mice displayed advanced adenocarcinomas, while heterozygous MRPL12 knockout mice showed atypical adenomatous hyperplasia, small adenomas, and low-grade adenocarcinomas. Homozygous MRPL12 knockout mice exhibited slight atypical adenomatous hyperplasia and structural disturbance (Fig. 1E). These results indicate that MRPL12 absence significantly impedes tumor progression in KP mice. Immunohistochemical (IHC) analysis of Ad-Cre-treated KP mice showed that MRPL12 expression was upregulated in tumor tissue compared to para-cancerous tissue (Fig. 1F and G). We further analyzed the relationship between KRAS or Tp53 and MRPL12 expression in LUAD tissues. Our analysis revealed that MRPL12 expression was significantly higher in KRAS mutant LUAD tissues compared to non-KRAS mutant tissues (Fig. S1A). However, no significant correlation was observed between MRPL12 expression and TP53 status (Fig. S1B).

Subsequently, we evaluated the impact of MRPL12 ablation on the overall survival of KP mice by comparing survival probabilities between the two groups. As depicted in Fig. 1H, Ad-Cre-treated KP mice had a median survival time of approximately 115 days. In contrast, MRPL12 knockout extended the median survival time to about 135 days, indicating that the reduced tumor burden resulting from MRPL12 deletion indeed improved survival. Collectively, these findings underscore the critical role of MRPL12 in LUAD development.

### *MRPL12* is highly expressed in LUAD organoid, tissues, and cells and associated with poor survival

**Fig. 2:**
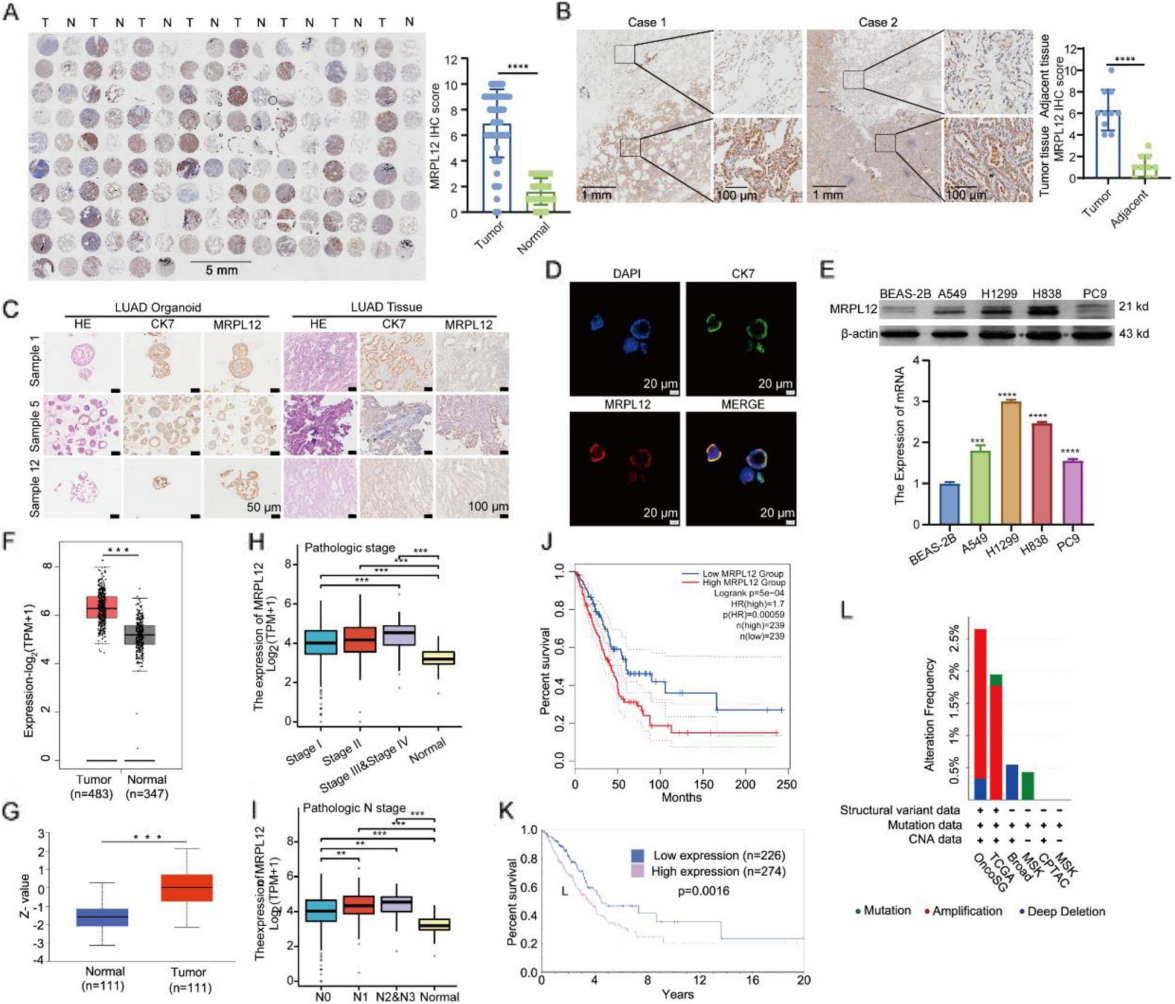
MRPL12 are overexpressed in LUAD organoid, tissues, and cells and associated with poor survival. **A** IHC analysis of MRPL12 was conducted on 75 pairs of tissues from patients with LUAD and their corresponding adjacent tissues. **B** Representative IHC images illustrating MRPL12 expression in tissues from patients with LUAD. **C** HE staining and IHC analysis of Cytokeratin 7 (CK7) and MRPL12 in human LUAD patient-derived organoids (PDO) compared to their corresponding original LUAD tissues. **D** Immunofluorescence (IF) analysis of CK7 and MRPL12 in PDO. Scale bar: 20 μm. **E** Analysis of MRPL12 expression levels in LUAD cell lines using Western blotting and RT-PCR. **F** The mRNA level of MRPL12 in LUAD was analyzed in GEPIA2.** G** Analysis of MRPL12 protein levels in LUAD using the UALCA. **H**,** I** Investigation of the correlation between MRPL12 expression levels and T, N, M, and pathological stage using TCGA datasets. **J**, **K** Assessment of overall survival in patients with high MRPL12 mRNA (**J**) or protein (**K**) levels. **L** Mutation analysis of the MRPL12 gene in LUAD based on the cBioPortal database. **, *p* < 0.01; ***, *p* < 0.001; ****, *p* < 0.0001

To further elucidate the role of MRPL12 in LUAD, we initially examined MRPL12 expression in LUAD using tissue microarrays. Analysis revealed a significant upregulation of MRPL12 in LUAD tissues compared to normal lung tissues (Fig. 2A). Additionally, clinical LUAD tissues exhibited markedly higher MRPL12 expression than their paracancerous counterparts (Fig. 2B). Human LUAD PDO, serving as faithful representations of original tumors, were constructed (Fig. S1C) and demonstrated elevated MRPL12 expression, validated by HE staining and CK7 immunostaining, a LUAD marker [32]. Notably, MRPL12 expression is relatively high in PDO, showing a pattern similar to that of CK7 (Fig. 2C and D). Further analysis of LUAD cells (A549, H1299, H838, and PC9) confirmed upregulated protein and mRNA levels of MRPL12 (Fig. 2E). Validation of these results was pursued through TCGA database analysis. Both GEPIA2 (http://gepia2.cancer-pku.cn/#index) and UALCAN (https://ualcan.path.uab.edu/cgi-bin/ualcan-res.pl) bioinformatics tools indicated higher mRNA levels of MRPL12 in LUAD tissues compared to normal tissues (Fig. 2F and Fig. S1D). Correspondingly, the protein level of MRPL12 was elevated in LUAD tissues (Fig. 2G). Survival analysis using TCGA databases showed higher MRPL12 expression in deceased LUAD patients than in living patients (Fig. S1E). MRPL12 expression correlated significantly with advanced pathologic stages (III and IV) and demonstrated a positive association with higher T, M, and particularly N stages (Fig. 2H, I, and Fig. S1F). These results collectively affirm the upregulation of both MRPL12 mRNA and protein levels in LUAD tissues. Furthermore, the association between high MRPL12 expression and poor prognosis in LUAD patients was explored using various online databases. Patients with elevated MRPL12 mRNA or protein levels exhibited poorer overall survival (Fig. 2J and K, Fig. S1G). Conversely, higher MRPL12 expression in lung squamous cell carcinoma did not correlate with poor prognosis (Fig. S1H). Analysis of lung cancer mutation data from the cBioportal database indicated infrequent MRPL12 gene mutations in LUAD, with a frequency of less than 2.5% (Fig. 2L). These findings underscore MRPL12 as upregulated in LUAD and a potential predictor of poor survival in LUAD patients.

### *MRPL12* facilitates LUAD tumorigenesis in vitro and in vivo

**Fig. 3:**
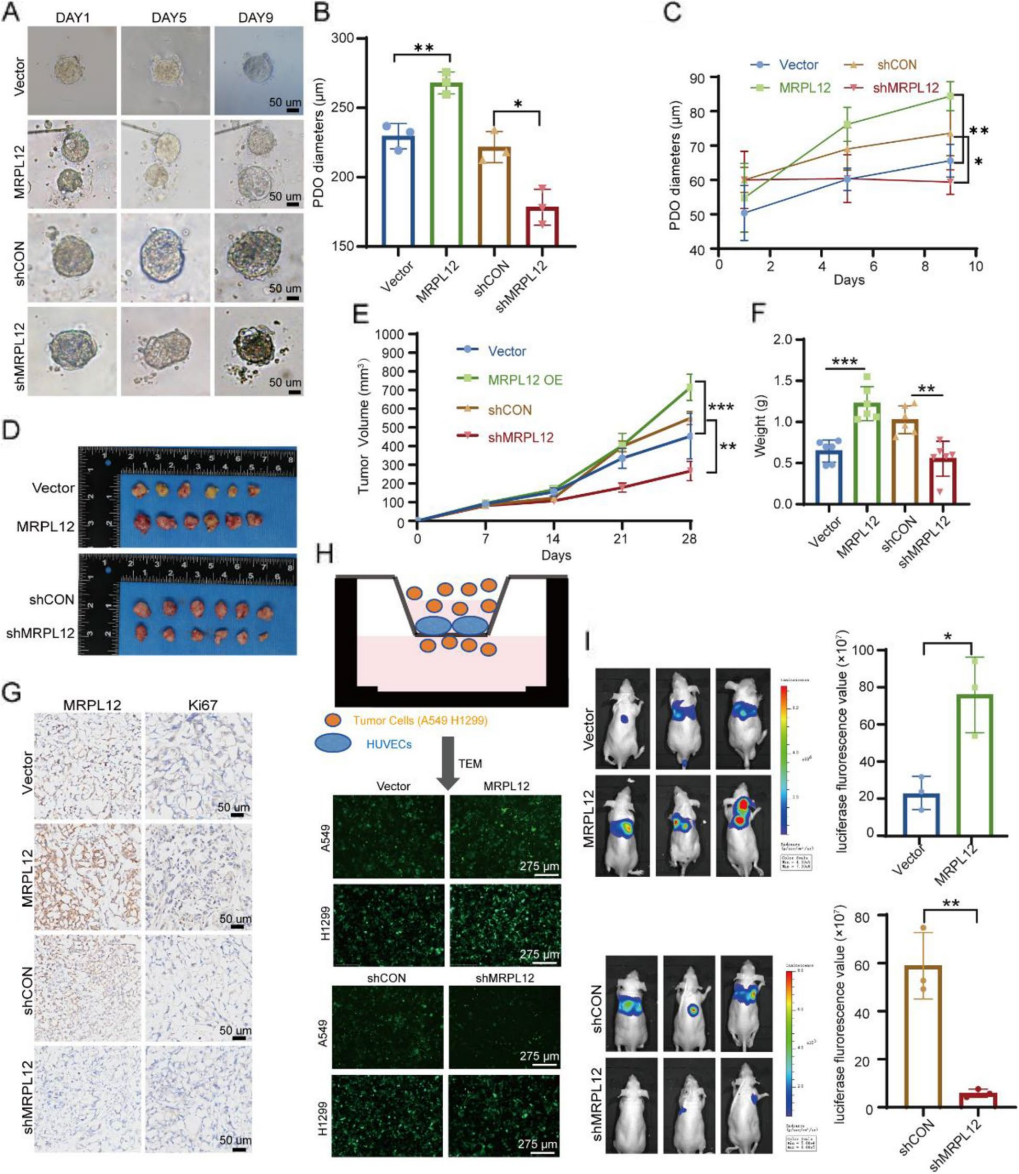
MRPL12 facilitates LUAD tumorigenesis. **A** Images depicting organoids subjected to MRPL12 overexpression and knockdown conditions. Scale bar: 50 μm. **B**, **C** Quantification of diameters of transfected organoids. **D** Analysis of A549 cell-derived xenografts with stable MRPL12 expression (*n* = 6). **E**, **F** Quantification of tumor weights and volumes in the xenografts (*n* = 6). **G** Representative IHC staining of MRPL12 and Ki67 in xenograft tissues. Scale bar: 50 μm. **H** Trans-endothelial migration assays illustrating HUVEC cell-coated inserts with A549 and H1299 tumor cells on top. Transmigration of tumor cells to the bottom was measured after 24 h. **I** Left: Fluorescence intensity detection in mice for in vivo metastasis evaluation. Right: Corresponding statistical analysis (*n* = 3). *, *p* < 0.05; **, *p* < 0.01; ***, *p* < 0.001

To elucidate the biological functions of MRPL12 in LUAD, we generated stable MRPL12 overexpression and knockdown cell lines in A549 and H1299 cells using a lentiviral delivery system (Fig. S1I). As depicted in Fig. S2A and S2B, MRPL12 knockdown significantly attenuated anchorage-independent and anchorage-dependent proliferation in A549 and H1299 cells, whereas MRPL12 overexpression enhanced LUAD cell proliferation. Further assessment using CRISPR/Cas9 technology to knock out MRPL12 in A549 and H1299 cells demonstrated a substantial inhibition of LUAD cell proliferation, and re-overexpression of MRPL12 restored the proliferative phenotype (Fig. S2C). PDO, mirroring tumor characteristics, are valuable for predicting responses to cancer therapies [33–35]. To corroborate MRPL12’s regulatory role in LUAD cell proliferation, we analyzed its effects on PDO formation. Lentivirus-mediated MRPL12 knockdown constrained PDO growth compared to control lentivirus-transfected organoids, while overexpression facilitated organoid formation (Fig. S1J), Fig. 3A, B and C). We further validated MRPL12’s role in LUAD cell proliferation in vivo through a xenograft experiment. A549 cells with stable MRPL12 overexpression or knockdown were subcutaneously injected into nude mice. Remarkably, MRPL12 knockdown led to a significant reduction in tumor growth, evident from decreased tumor volume and weight (Fig. 3D, E and F). Additionally, Ki67 expression in xenografts with MRPL12 knockdown were significantly decreased compared to controls, whereas MRPL12 overexpression exhibited the opposite pattern (Fig. 3G). These results underscore MRPL12’s involvement in LUAD cell proliferation.

To investigate the regulatory role of MRPL12 in LUAD metastasis, transwell migration and invasion assays were employed to assess the motility of LUAD cells. MRPL12 knockdown significantly suppressed the migration and invasion of A549 and H1299 cells, while MRPL12 overexpression promoted these processes (Fig. S2D). To further verify the role of MRPL12 in LUAD cells migration and invasion, we knockout MRPL12 using sgRNA. We found that MRPL12 knockout significantly suppressed the migration and invasion ability of A549 and H1299 cells and MRPL12 re-expression restored these capabilities (Fig. S2E). As trans-microvascular endothelial migration (TEM) is a crucial step in tumor metastasis [36], we explored MRPL12’s role in the transmigration of LUAD cells through microvascular endothelium. In the transmigration assay, human umbilical vein endothelial cells (HUVEC) pre-coated transwell chambers, to which A549 and H1299 cells were added, displayed significantly increased trans-endothelial migratory ability with MRPL12 overexpression and decreased ability with MRPL12 knockdown after 24 h of co-culture (Fig. 3H and S2F). To examine MRPL12’s effects on distant metastasis in vivo, MRPL12-overexpressing or -knockdown A549 cells were injected into the tail vein of nude mice. Lung metastasis signals were markedly stronger in the MRPL12-overexpression group and weaker in the MRPL12-knockdown group (Fig. 3I). Ingenuity Pathway Analysis (IPA) of diseases and function demonstrated that differentially expressed genes after MRPL12 knockdown in A549 cells were involved in cellular movement (Fig. S2G). Additionally, the extracellular matrix organization pathway related to cell migration and invasion was enriched (Fig. S2H). Co‐expression analysis combined with KEGG enrichment analysis can be used to reliably predict the potential biological functions of target genes [37]. Genes co-expressed with MRPL12 were mainly associated with the cell cycle, cell adhesion molecules, ECM-receptor interaction, and focal adhesion (Fig. S3A). We further observed that MRPL12 overexpression and knockdown influenced the expression of CCNB2, N-Cad, E-Cad, and p-FAK to varying degrees (Fig. S3B), suggesting that MRPL12 plays a role in regulating biological processes such as cell proliferation and metastasis. These results collectively indicate that MRPL12 plays a crucial role in promoting proliferation and metastasis in LUAD, suggesting its involvement in LUAD tumorigenesis both in vitro and in vivo.

### *MRPL12* promotes lung tumorigenesis via promoting mitochondrial oxidative phosphorylation

**Fig. 4:**
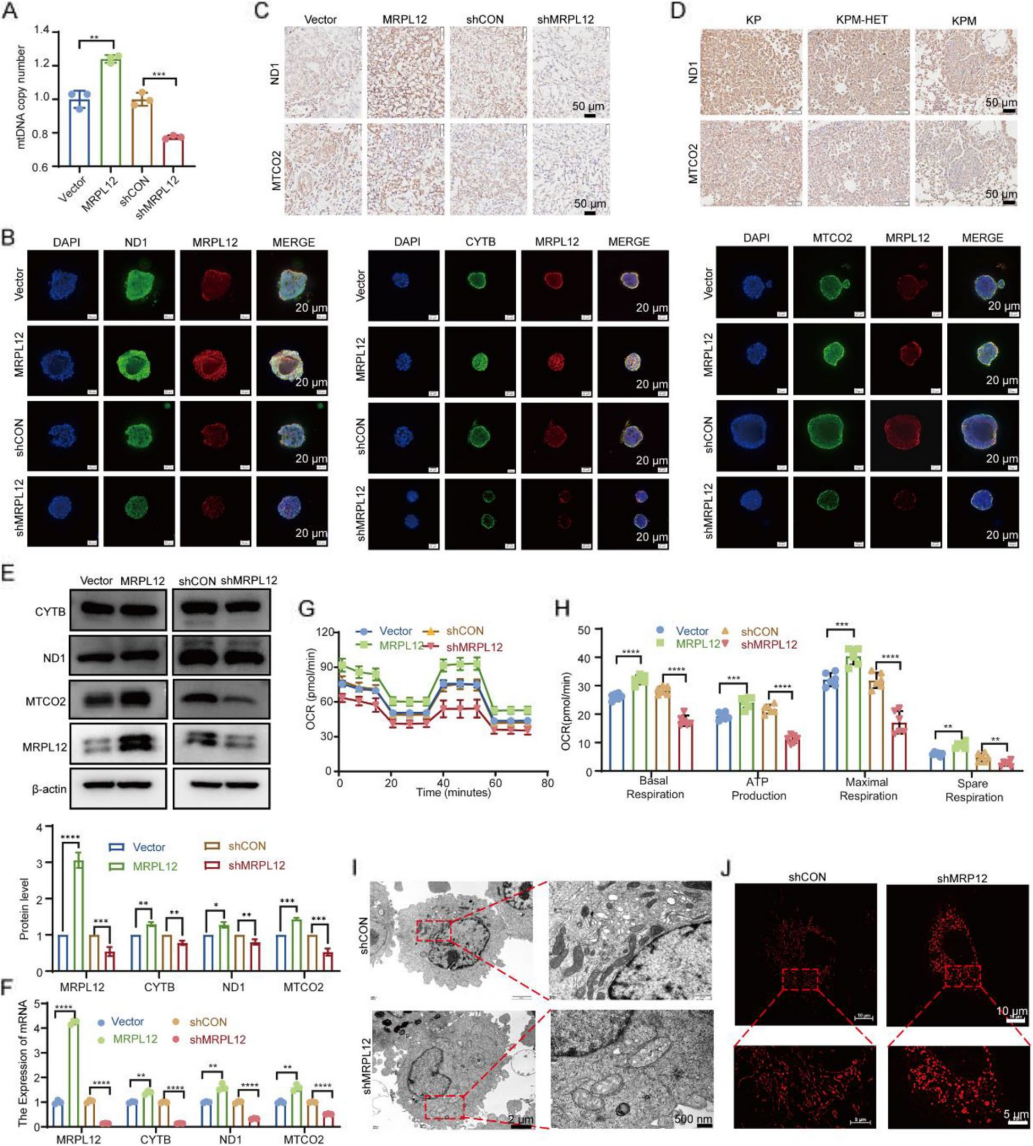
MRPL12 promotes lung tumorigenesis via promoting mitochondrial oxidative phosphorylation. **A** Analysis of mitochondrial DNA (mtDNA) copy number in A549 cells subjected to MRPL12 knockdown or overexpression. **B** IF analysis of OXPHOS-related genes and MRPL12 in organoids post-transfection.** C**, **D** IHC examination of ND1 and MTCO2 in xenografts and lung tissues derived from genetically engineered mice. **E**, **F** Evaluation of protein and mRNA levels of OXPHOS complexes in A549 cells with MRPL12 knockdown or overexpression. **G** Measurement of oxygen consumption rate (OCR) in A549 cells with MRPL12 knockdown or overexpression using Seahorse XFe96. **H** Analysis of mitochondrial OXPHOS, including basal respiration, maximal respiration, ATP production, and spare respiratory capacity in MRPL12 knockdown or overexpressed A549 cells. **I** Electron microscopy analysis of A549 cells for observing mitochondrial morphology. **J** Three-dimensional Structured Illumination Microscopy (SIM) images of mitochondria stained with MitoTracker Red CMXRos. *, *p* < 0.05; **, *p* < 0.01; ***, *p* < 0.001; ****, *p* < 0.0001

MRPL12, recognized as a mitochondrial ribosomal protein, has been reported to participate not only in the translation of mitochondrial proteins but also in the regulation of mitochondrial biogenesis as a transcription factor [16, 17]. Our previous research emphasized the pivotal role of MRPL12 in modulating mitochondrial OXPHOS and metabolism in various metabolic diseases, including diabetes [18, 19]. KEGG enrichment analysis of genes co-expressed with MRPL12 also highlighted its association with OXPHOS and metabolism (Fig. S3A). However, the involvement of MRPL12 in the development of LUAD through the regulation of mitochondrial function remains unclear. In this study, MRPL12 overexpression or knockdown in A549 and H1299 cells resulted in an increase or decrease in mtDNA copy numbers (Fig. 4A and S4A). Then, MRPL12 was knocked down or overexpressed in organoids (Fig. S1J). It was found that MRPL12 influences the expression levels of mitochondria-encoded oxidative respiratory chain complex-related genes, including ND1, CYTB, and MTCO2, in LUAD PDOs (Fig. 4B). Additionally, xenograft tissues of mice exhibited altered expression of MTCO2 and ND1 in response to MRPL12 knockdown or overexpression (Fig. 4C). Notably, MRPL12 knockout in the Tp53^fl/fl^;Kras^G12D^ mouse model significantly reduced the expression of ND1 and MTCO2 (Fig. 4D). Both mRNA and protein levels of these mitochondria-encoded genes in A549 and H1299 cells showed consistent trends after MRPL12 overexpression and knockdown (Fig. 4E, F and S4B, S4C). Functional assessments using a Seahorse XFe96 extracellular flux analyzer revealed that MRPL12 overexpression significantly enhanced spare respiratory capacity, basal and maximal respiratory capacity, and OCR coupled to ATP synthesis, while MRPL12 knockdown had the opposite effect (Fig. 4G, H and S4D, S4E). Also, we found that mitochondrial mass decreased after MRPL12 knockdown and increased after MRPL12 overexpression (Fig. S4F). Furthermore, MRPL12’s impact on mitochondrial morphology was assessed using structured illumination super-resolution microscopy (SIM) and electron microscopy, revealing that MRPL12 knockdown induced structural damage in mitochondria, including swelling, irregular arrangement, less recognizable cristae, and reduced volume and abundance. Notably, these aberrations were partially restored upon MRPL12 re-overexpression (Fig. 4I, J, 7F, and G).

The above results revealed that MRPL12 is required for mitochondrial homeostasis, especially the regulation of mitochondrial respiration. Crucially, the proliferation, migration, and invasion effects induced by MRPL12 overexpression in LUAD cells were effectively suppressed when exposed to a mitochondrial aerobic respiration inhibitor (Fig. S4G and S4H), suggesting that MRPL12 regulates LUAD cells’ proliferation, migration, and invasion through the manipulation of OXPHOS. Collectively, these results underscore the essential role of MRPL12 as a regulator of mitochondrial energy metabolism, potentially promoting LUAD tumorigenesis.

### UBASH3B interacts with MRPL12

**Fig. 5:**
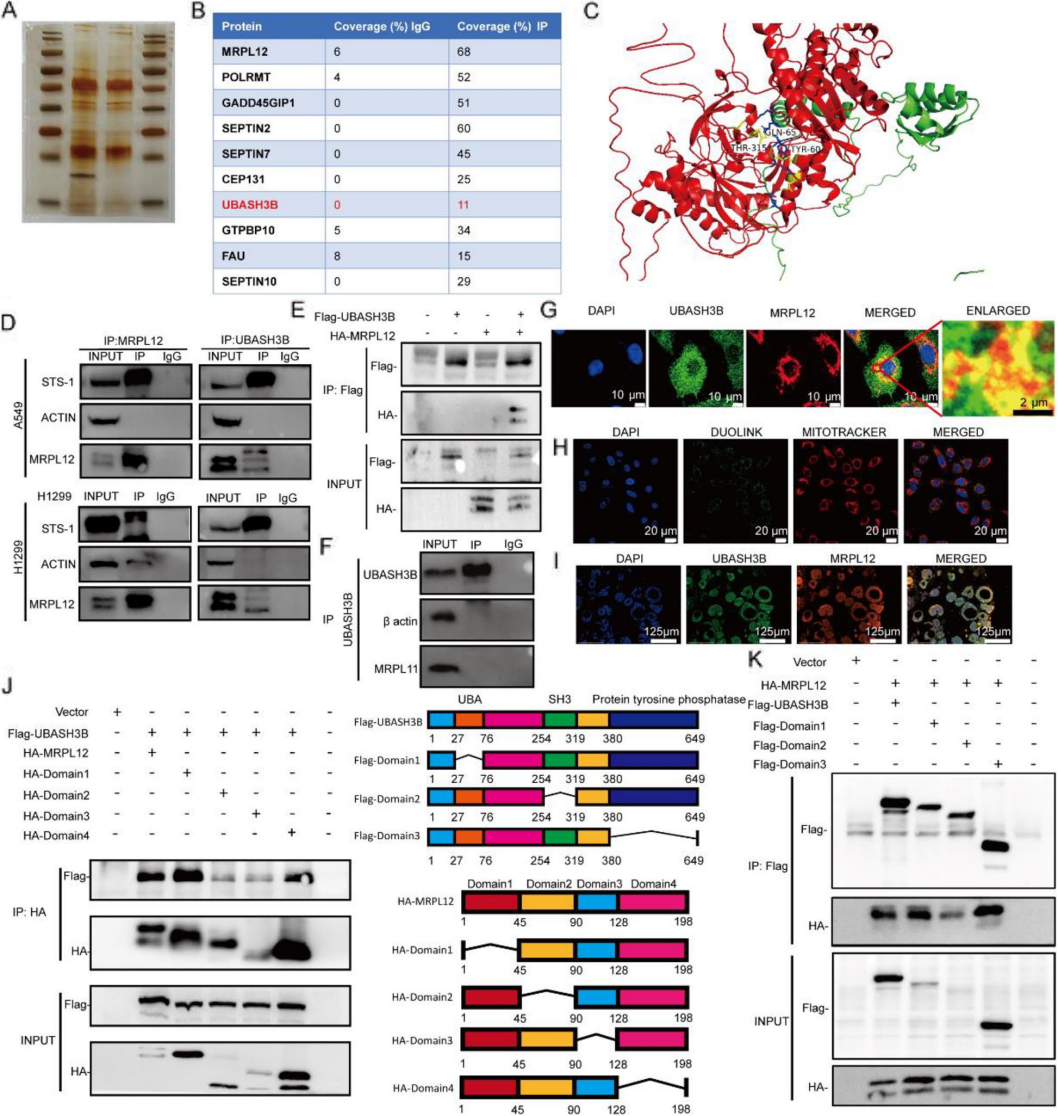
UBASH3B interacts with MRPL12. **A** Co-immunoprecipitation (Co-IP) of MRPL12 complexes, followed by the identification of MRPL12-binding proteins through combined silver staining and mass spectrometry (MS). **B** A tabulated summary of mass spectrometry results, delineating the bait protein MRPL12 and its identified binding partners. **C** Molecular docking of 3D structures demonstrating the interaction between MRPL12 and UBASH3B.** D** Co-IP assay illustrating the interaction between MRPL12 and UBASH3B in A549 and H1299 cells. **E** Co-IP assays revealing the interaction between MRPL12 and UBASH3B in HEK293T cells. **F** Co-IP assay investigating the interaction between MRPL11 and UBASH3B in A549 cells. **G** IF staining of endogenous MRPL12 (red) and UBASH3B (green) in A549 cells, with nuclei counterstained using DAPI (blue). **H** Visualization of MRPL12 and UBASH3B interaction in A549 cells using the Duolink proximity ligation assay. **I** IF staining for MRPL12 (red) and UBASH3B (green) localization in LUAD PDO. **J**, **K** Schematic diagrams and IP analyses demonstrating the interaction between MRPL12-UBASH3B in HEK293T cells. Cells were transfected with HA-MRPL12 and Flag-UBASH3B or various mutant constructs

The preceding findings indicate that MRPL12 plays a role in LUAD development through the regulation of mitochondrial OXPHOS, implying its potential as an intervention target for LUAD treatment. To our understanding, interventions at the transcriptional level frequently lack specificity, whereas interventions at the protein modification level offer notable specificity and targeting capabilities. This approach holds the potential to markedly diminish side effects and carries crucial clinical application value. Consequently, our subsequent focus centers on the post-transcriptional modification of MRPL12, aiming to identify potential intervention targets and furnish effective strategies for clinical intervention in LUAD. Initially, co-immunoprecipitation (co-IP) and mass spectrometry (MS) were conducted to identify potential interacting proteins of MRPL12 in A549 cells (Fig. 5A). The results revealed an interaction between MRPL12 and UBASH3B, a protein tyrosine phosphatase involved in post-transcriptional modification [38] (Fig. 5B). Molecular docking on the ZDOCK database (http://zdock.umassmed.edu/) supported the formation of stable complexes between MRPL12 and UBASH3B based on their compatible protein spatial structures (Fig. 5C). IP assays further confirmed this interaction in both A549 and H1299 cells (Fig. 5D). Ectopic expression of MRPL12 and UBASH3B in 293 T cells validated these results (Fig. 5E). Moreover, an endogenous co-IP assay demonstrated that UBASH3B specifically interacts with MRPL12, not MRPL11 (Fig. 5F). Immunofluorescence (IF) staining depicted co-localization of MRPL12 and UBASH3B (Fig. 5G). The proximity ligation assay (PLA) further substantiated the interaction between MRPL12 and UBASH3B (Fig. 5H). Crucially, this interaction was affirmed in organoids (Fig. 5I). Further delineation of the protein segment mediating the MRPL12-UBASH3B interaction was achieved through co-IP experiments, indicating that the segment (aa 45–90) of MRPL12 interacts with UBASH3B (Fig. 5J). The structure of UBASH3B encompasses interactive domains UBA, SH3, and the histidine phosphatase enzymatic domain. UBASH3B (Δ254–319), lacking the SH3 domain, failed to interact with MRPL12, implicating the SH3 domain of UBASH3B as the binding segment for MRPL12 (Fig. 5K). In summary, UBASH3B, through its SH3 domain, emerges as a partner of MRPL12 in a specific interaction.

### UBASH3B dephosphorylates MRPL12 at residue Y60 and inhibits lung tumorigenesis

**Fig. 6:**
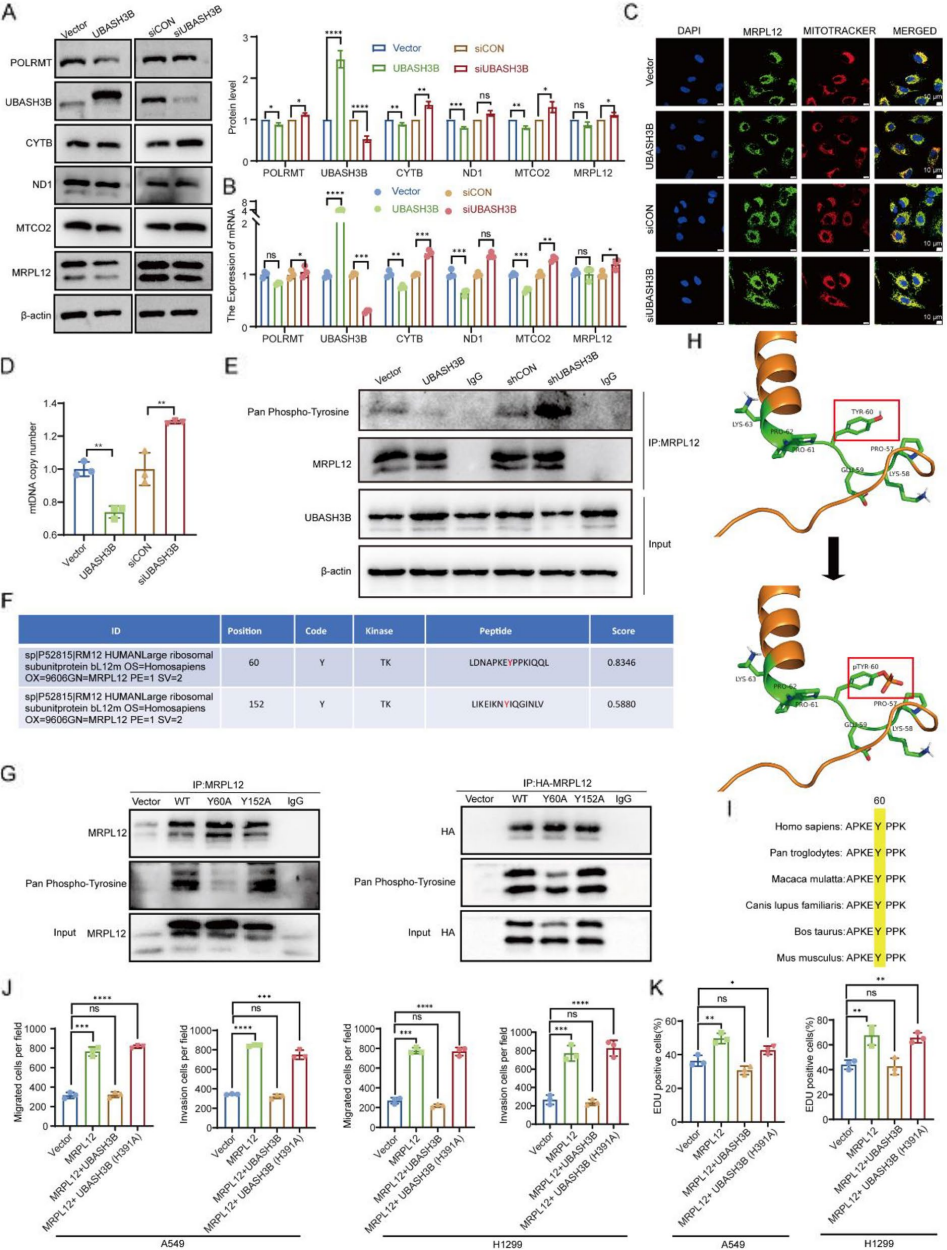
UBASH3B dephosphorylates MRPL12 at residue Y60 and inhibits lung tumorigenesis. **A-B** Evaluation of protein and mRNA levels of OXPHOS complexes in A549 cells subjected to UBASH3B knockdown or overexpression. **C** IF analysis depicting MRPL12 localization in A549 cells under conditions of UBASH3B knockdown or overexpression.** D** mtDNA copy number assessment in A549 cells with altered UBASH3B expression. **E** Analysis of MRPL12 phosphorylation levels in A549 cells with varying UBASH3B expression. **F** Prediction of MRPL12 phosphorylation sites utilizing the Group-based Prediction System (GPS). **G** Immunoprecipitation analysis in A549 and HEK293T cells transfected with HA-MRPL12 WT (wild type), HA-MRPL12 Y60A (Y60 mutant), and MRPL12 Y152A (Y152 mutant) for 48 h. **H** Illustration of structural changes in MRPL12 following phosphorylation. **I** Highlighting the high conservation of amino acids adjacent to MRPL12 Y60 in diverse species. **J** Quantification of migration and invasion ability of A549 and H1299 cells after overexpression of MRPL12 with UBASH3B WT or UBASH3B H391A mutant in transwell assays. **K** Quantification of proliferation ability of A549 and H1299 cells after overexpression of MRPL12 with UBASH3B WT or UBASH3B H391A mutant in EDU assays. *, *p* < 0.05; **, *p* < 0.01; ***, *p* < 0.001; ****, *p* < 0.0001; ns, *p* > 0.05

Initially, we investigated whether the interaction between UBASH3B and MRPL12 influences the protein stability of MRPL12. The data presented in Fig. 6A indicate that MRPL12 protein levels remained relatively unchanged upon UBASH3B knockdown or overexpression in A549 and H1299 cell lines. Subsequently, we confirmed that UBASH3B exerted no discernible impact on the mitochondrial localization of MRPL12 (Fig. 6C). Given the established association of MRPL12 with mitochondrial biosynthesis in LUAD cells, we postulated that UBASH3B might also play a role in the regulation of mitochondrial function. To test this hypothesis, we modulated UBASH3B expression in LUAD cells. Results revealed that UBASH3B knockdown led to an upregulation in the protein levels of ND1, CYTB, and MTCO2, whereas UBASH3B overexpression resulted in their downregulation (Fig. 6A). Consistent alterations were observed at the mRNA level for mitochondria-encoded genes (Fig. 6B). Furthermore, overexpression or knockdown of UBASH3B influenced the mitochondrial copy number, indicating a potential role in the regulation of mitochondrial biosynthesis (Fig. 6D). These findings suggest that UBASH3B may modulate mitochondrial biosynthesis by binding to MRPL12, influencing MRPL12’s biological function rather than its protein stability and subcellular localization. Notably, UBASH3B, known for its tyrosine protein phosphatase activity, has been implicated as an oncogenic driver in triple-negative breast cancer invasion and metastasis [39]. Subsequent experiments were conducted to ascertain whether UBASH3B could impact the phosphorylation modification level of MRPL12. The results demonstrated that the phosphorylated MRPL12 level decreased or increased upon overexpression or knockdown of UBASH3B (Fig. 6E). Two tyrosine phosphorylation sites of MRPL12, Y60 and Y152, were identified through bioinformatics (Fig. 6F). Subsequently, mutant plasmids were constructed for MRPL12 Y60A and MRPL12 Y152A to mimic dephosphorylation, and their transfection revealed a significant reduction in the tyrosine phosphorylation level of MRPL12 after Y60A mutation, while Y152A mutation did not significantly alter the tyrosine phosphorylation level, suggesting Y60 as the primary tyrosine phosphorylation site of MRPL12 (Fig. 6G). We then mapped the protein structure of MRPL12 before and after phosphorylation (Fig. 6H). We also found that MRPL12 Y60 is evolutionarily conserved among various species (Fig. 6I).

UBASH3B’s involvement in the pathogenesis of various tumors, including breast cancer and leukemia, has been documented [39, 40]. However, its role and mechanisms in lung cancer remain unexplored. Initial analysis using the TIMER2 online platform (http://timer.cistrome.org/) revealed that, in contrast to normal tissue, UBASH3B expression was upregulated in most tumors but downregulated in LUAD (Fig. S5A). Further scrutiny of a different LUAD TCGA dataset confirmed a significant downregulation of UBASH3B in LUAD tissues compared to para-carcinoma or matched adjacent tissues (Fig. S5B, S5C, and S5D). Similarly, IHC of clinical tissues showed that UBASH3B expression was significantly lower in LUAD tissues compared to adjacent tissues (Fig. S6A). Additionally, the expression level of UBASH3B in organoids was lower than that of CK7 (Fig. S6A). We also analyzed the relationship between UBASH3B expression and pathological grade or TNM stage progression (Fig. S6B and S6C). Examination of various cohort databases indicated that low UBASH3B expression correlates with poor overall survival in LUAD patients (Fig. S5E and 5F), suggesting a potential tumor-suppressive role for UBASH3B in LUAD. To validate these findings, we manipulated UBASH3B expression in A549 and H1299 cell lines, observing enhanced cell proliferation, migration, and invasion following UBASH3B knockdown, and the opposite effect upon overexpression (Fig. S5G and S5H). These results suggest that UBASH3B, by interacting with MRPL12 and regulating its tyrosine phosphorylation level, influences mitochondrial function. Consequently, we hypothesized that UBASH3B may impact the MRPL12-mediated malignant phenotype in LUAD. Indeed, UBASH3B overexpression reversed the proliferation, migration, and invasion induced by MRPL12 overexpression in LUAD cells (Fig. 6J, 6K, S5I and S5J). Conversely, the UBASH3B H391A mutant, an inactivated tyrosine phosphatase, failed to reverse the tumor malignant phenotype caused by MRPL12 overexpression (Fig. 6J, 6K and S5I and 5 J). To further elucidate the role of UBASH3B in modulating the malignant phenotype of LUAD cells via regulation of MRPL12 Y60 phosphorylation, we introduced a mutation substituting tyrosine (Y) at position Y60 in MRPL12 with glutamic acid (E) to mimic phosphorylated Y60. We found that overexpression of UBASH3B inhibited the tumor phenotype induced by MRPL12 WT overexpression but did not affect the tumor phenotype induced by MRPL12 Y60E overexpression (Fig. S6D and S6E). Further analysis revealed that UBASH3B knockdown exacerbated the LUAD cell phenotype caused by MRPL12 WT overexpression but had no effect on the phenotype induced by MRPL12 Y60A overexpression. These results suggest that UBASH3B exerts its effects primarily through the MRPL12 Y60 site (Fig. S6D and S6E). Overall, these findings suggest that UBASH3B, through its tyrosine phosphatase activity, regulates the Y60 phosphorylation of MRPL12, contributing to the development of LUAD.

### MRPL12 Y60 dephosphorylation attenuates mitochondrial biosynthesis via affecting the binding of MRPL12 and POLRMT

**Fig. 7:**
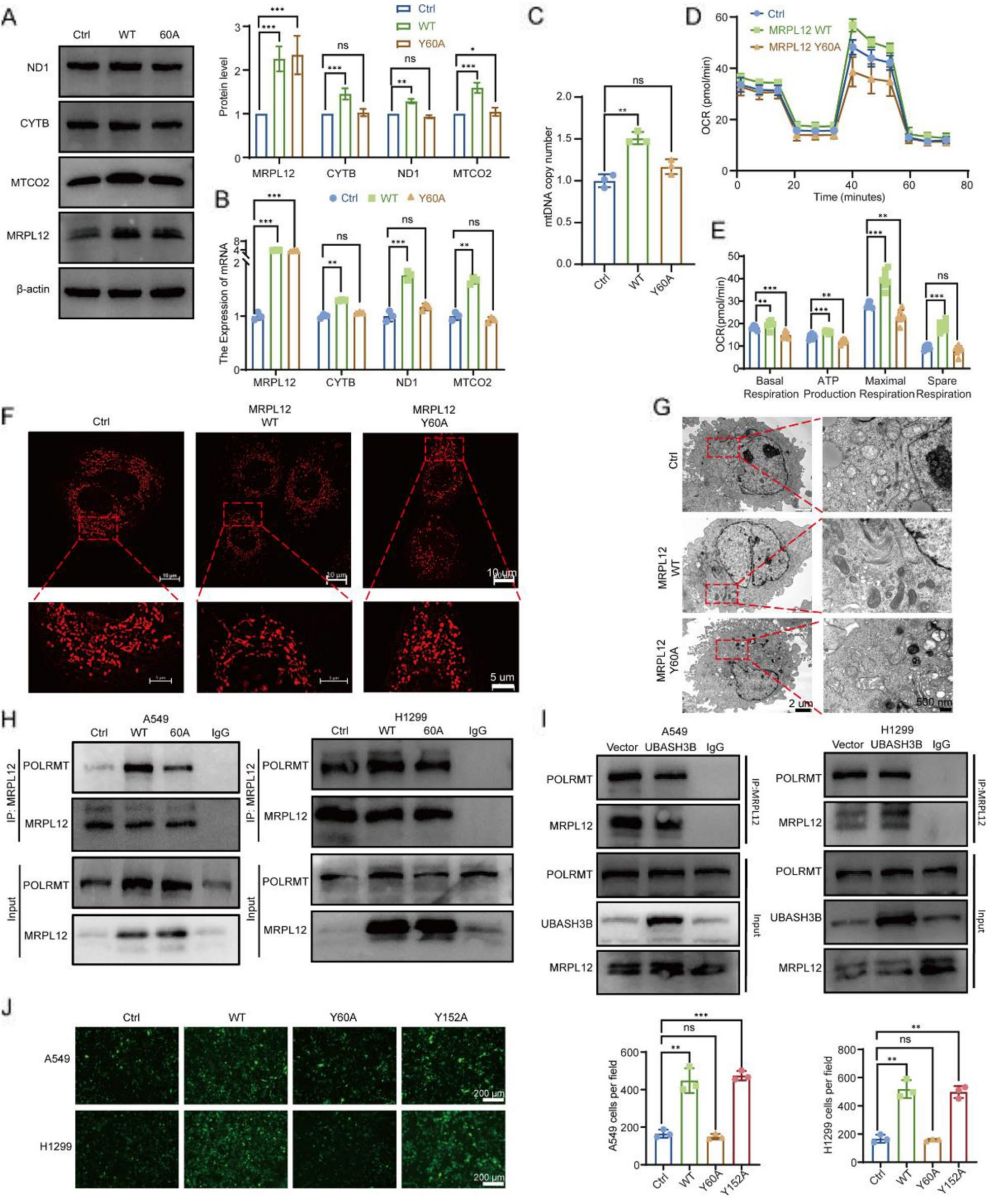
MRPL12 Y60 dephosphorylation attenuates mitochondrial biosynthesis via affecting the binding of MRPL12 and POLRMT. **A**,** B** Assessment of protein and mRNA levels of OXPHOS complexes in A549 cells expressing either MRPL12 WT or MRPL12 Y60A mutation plasmid. **C** Quantification of mtDNA copy number in A549 cells overexpressing MRPL12 Y60A or MRPL12 WT. **D** Analysis of OCR in A549 cells with MRPL12 Y60A or MRPL12 WT overexpression using Seahorse XFe96. **E** Quantification of basal respiration, ATP production, maximal respiration, and spare respiratory capacity, respectively. **F** Visualization of mitochondria in MRPL12-knockout A549 cells with re-overexpression of MRPL12 Y60A or MRPL12 WT, stained with MitoTracker Red CMXRos, and observed using SIM. **G** Electron microscopy analysis of mitochondrial morphology in MRPL12-knockout A549 cells reexpressing MRPL12 Y60A or MRPL12 WT. **H**, **I** Co-IP assays in A549 and H1299 cells lysed and immunoprecipitated with MRPL12 antibody, followed by western blot analysis with POLRMT. **J** Representative immunofluorescence images and quantitation of A549 and H1299 cells overexpressing MRPL12 WT, MRPL12 Y60A, or MRPL12 Y152A plasmid in trans-endothelial migration assays. *, *p* < 0.05; **, *p* < 0.01; ***, *p* < 0.001, ns, *p* > 0.05

The aforementioned findings imply a direct correlation between the phosphorylation level of MRPL12 Y60 and the functional role of MRPL12, suggesting its pivotal involvement in LUAD tumorigenesis. Despite this, the functional implications of Y60 phosphorylation on MRPL12 have not been delineated. Unlike MRPL12 overexpression, which elevated mRNA and protein levels of mitochondrial coding genes (e.g., CYTB, MTCO2, and ND1), the MRPL12 Y60A mutant failed to induce expression of these genes (Fig. 7A, B, and S7A, S7B). Furthermore, the MRPL12 Y60A mutant demonstrated an inability to upregulate mtDNA copy numbers (Fig. 7C and S7C). In consistency, MRPL12 overexpression significantly increased basal oxygen consumption rate (OCR), ATP-linked OCR, maximal respiration, and spare respiratory capacity. In contrast, the MRPL12 Y60A mutant lacked the capacity to enhance mitochondrial OXPHOS (Fig. 7D, E, and S7D, S7E). These results indicate that the phosphorylation of MRPL12 Y60 is directly linked to its ability to regulate mitochondrial biosynthesis—specifically, a decrease in the Y60 phosphorylation level correlates with diminished mitochondrial biosynthetic capacity mediated by MRPL12.

To delve further into the impact of MRPL12 on mitochondrial morphology and structure, we overexpressed MRPL12 WT and MRPL12 Y60A after knocking out MRPL12. Knocking out MRPL12 resulted in mitochondrial swelling, reduced mitochondrial ridges, and structural disorder, while MRPL12 WT overexpression partially restored mitochondrial morphology. However, the overexpression of MRPL12 Y60A did not significantly improve mitochondrial structure and morphology (Fig. 7F, G). These outcomes underscore the significance of the phosphorylation level of MRPL12 Y60 as a crucial post-translational modification site governing the physiological function of MRPL12.

Given that MRPL12 can directly bind to and activate mitochondrial RNA polymerase (POLRMT) to regulate mitochondrial biosynthesis [15], we hypothesized that MRPL12 Y60 phosphorylation might impact mitochondrial function by influencing MRPL12’s binding to POLRMT. As anticipated, the MRPL12 Y60A mutant attenuated the interaction between endogenous MRPL12 and POLRMT (Fig. 7H). However, the MRPL12 Y152A mutant did not affect the binding of MRPL12 to POLRMT (Fig. S7F). Additionally, UBASH3B overexpression weakened the binding of MRPL12 to POLRMT (Fig. 7I). These findings underscore the pivotal role of MRPL12 Y60 phosphorylation as a key modification site influencing the interaction between MRPL12 and POLRMT, consequently affecting mitochondrial function.

Based on the preceding experimental findings, we posited that the MRPL12 Y60A mutant might influence the malignant phenotype of LUAD cells. Indeed, we observed that MRPL12 WT overexpression enhanced the proliferation, migration, invasion, and trans-endothelial migration of A549 and H1299 cells. Conversely, overexpression of the MRPL12 Y60A mutant abolished the capacity of MRPL12 to promote the malignant phenotype of LUAD cells (Fig. 7J, S7G and S7H). Additionally, we ascertained that the MRPL12 Y152A mutant exerted no discernible effect on the malignant phenotype of LUAD cells induced by MRPL12 overexpression (Fig. 7J, S7G and S7H). Consequently, these results underscore that UBASH3B-mediated MRPL12 Y60 dephosphorylation selectively inhibits MRPL12 binding to POLRMT, leading to mitochondrial dysfunction and the suppressed malignant phenotype of LUAD.

### MRPL12 Y60 dephosphorylation inhibits tumor formation, metastasis, and organoid formation

**Fig. 8:**
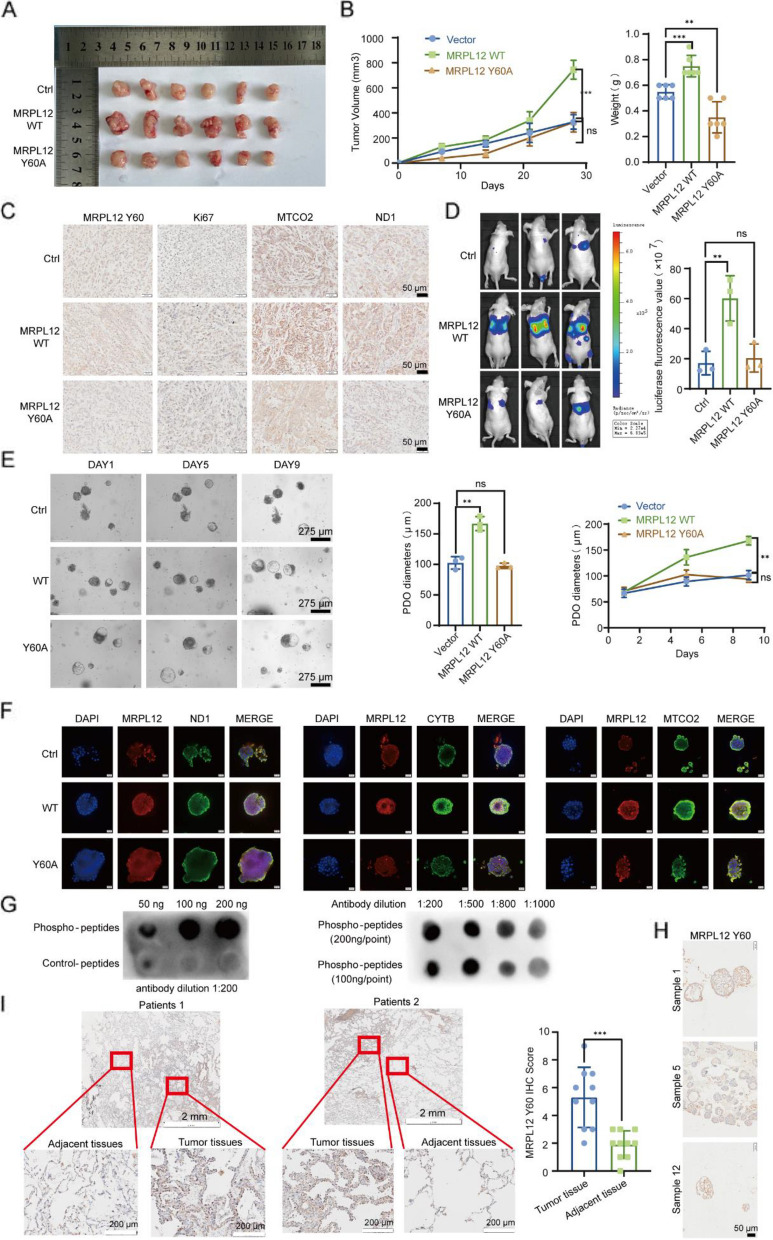
MRPL12 Y60 dephosphorylation inhibits tumor formation, metastasis, and organoid formation. **A** Analysis of xenografts derived from the A549 cell line with stable expression of MRPL12 WT or MRPL12 Y60A mutant (*n* = 6). **B** Quantification of tumor weights and volumes (*n* = 6). **C** Representative IHC staining depicting the phosphorylation level of MRPL12 Y60 and the protein levels of Ki67, MTCO2, and ND1 in xenograft tissues. **D** Left: Measurement of fluorescence intensity in mice for in vivo metastasis evaluation. Right: Corresponding statistical analysis. **E** Visualization of organoids overexpressing the MRPL12 Y60A mutation or MRPL12 WT plasmid, with quantification of their diameters. **F** IF analysis of OXPHOS components in organoids post-transfection with MRPL12 WT or MRPL12 Y60A mutant. **G** Verification of the specificity of the customized phosphorylated antibody for MRPL12 Y60 using Dot Blotting. **H** IHC analysis of organoids using the customized phosphorylated antibodies for MRPL12 Y60. **I** IHC analysis of LUAD patient samples using the customized phosphorylated antibodies for MRPL12 Y60. **, *p* < 0.01; ***, *p* < 0.001; ns, *p* > 0.05

The above-presented data indicates a correlation between the phosphorylation of MRPL12 Y60 and mitochondrial dysfunction, impacting LUAD tumorigenesis in vitro. Consequently, we substantiated the pivotal role of MRPL12 Y60 through validation in LUAD PDO, murine models, and clinical tissues. The xenograft tumor from the MRPL12 WT overexpression group exhibited significantly larger size and weight compared to the control group. In contrast, no significant changes in tumor size and weight were observed in the MRPL12 Y60A overexpression group compared to the control group (Fig. 8A and B). Xenograft IHC results demonstrated that MRPL12 WT overexpression upregulated the expression of MTCO2, ND1, and Ki67, whereas MRPL12 Y60A mutant overexpression had no significant effect on the expression of these genes (Fig. 8C). Subsequently, MRPL12 WT overexpression significantly promoted lung metastasis of A549 cells, while overexpression of MRPL12 Y60A mutant did not significantly enhance the lung metastasis capacity of A549 cells (Fig. 8D). Furthermore, the role of MRPL12 Y60A mutant was validated in LUAD PDO models, where, in accordance with in vivo results, MRPL12 Y60A mutants lost their ability to promote PDO formation (Fig. 8E). Additionally, the overexpression of MRPL12 Y60A mutants in PDO did not significantly increase the expression of MTCO2, ND1, and CYTB (Fig. 8F). These findings underscore the crucial role of the phosphorylation activity of MRPL12 Y60 for its oncogenic role in promoting proliferation and metastasis in LUAD. To ascertain the role of MRPL12 Y60, we developed a rabbit polyclonal antibody (Ab) specifically targeting phosphorylated MRPL12 Y60. This antibody selectively recognized the Y60 phosphorylation peptide rather than the Y60 peptide fragment (Fig. 8G). Using this specific antibody, we detected MRPL12 Y60 phosphorylation levels in PDO (Fig. 8H). Importantly, we found that MRPL12 Y60 phosphorylation levels were higher in LUAD tumor tissues compared to adjacent tissues (F ig. 8I). Thus, the phosphorylation of MRPL12 Y60 emerges as a crucial modification influencing the biological function of MRPL12 and LUAD development. Targeting MRPL12 Y60 phosphorylation presents a viable therapeutic strategy to counteract oncogenic processes of LUAD.

## Discussion

MRPL12, the initial mitochondrial ribosomal protein identified in mammals, has recently been recognized for its pivotal role in mitochondrial biogenesis and OXPHOS [15]. However, the precise function of MRPL12 and the molecular mechanisms underlying its involvement in the onset and progression of LUAD remain incompletely understood. The *LSL-Kras*^*G12D/*+^*;LSL-p53*^*−/−*^ mouse model, frequently employed in investigating the molecular intricacies of LUAD development and exploring novel therapeutic strategies, was utilized in this study [41]. By crossing MRPL12^fl/fl^ with *Tp53*^*fl/fl*^*;Kras*^*G12D*^ mice, we generated *Tp53*^*fl/fl*^*;Kras*^*G12D*^*;MRPL12*^*fl/fl*^ (KPM) composite mice. We found that MRPL12 knockout significantly inhibited *Tp53*^*−/−*^*; Kras*^*G12D*^ mice tumors developed in the lung tissue and prolonged the survival of the mice. Elevated MRPL12 expression was also confirmed in LUAD tissues, correlating with an unfavorable clinical prognosis. Recently developed three-dimensional organoid culture systems, such as patient-derived organoids (PDO), offer a more representative environment, mimicking in vivo conditions, and are especially advantageous for studying LUAD [32, 42]. In this investigation, we successfully cultured LUAD tissue-derived organoids and confirmed heightened MRPL12 expression using immunofluorescence and immunohistochemistry. Also, we revealed that MRPL12 involved in proliferation of LUAD PDO. Moreover, our findings indicate that MRPL12 promotes the proliferation, migration, and invasion of LUAD cells. Therefore, the observed promotion of proliferation, invasion, migration, and transvascular endothelial cell migration by MRPL12 both in vitro and in vivo implies a crucial role for MRPL12 in the malignant progression of LUAD tumors.

Prior investigations have established MRPL12’s involvement in various diseases, such as diabetic kidney disease and hepatic cellular cancer, through the regulation of mitochondrial biogenesis [18, 43]. In normal cellular processes, ATP generation primarily occurs via OXPHOS on the inner mitochondrial membrane, facilitated by the electron transport chain and ATP synthase. However, cancer cells, even in oxygen-sufficient conditions, exhibit a predilection for energy production through glycolysis, a phenomenon recognized as the ‘Warburg Effect’ [44]. This metabolic shift enables cancer cells to thrive and proliferate in hypoxic environments, concurrently supplying biosynthetic materials essential for rapid growth. Notwithstanding, cancer cells do not entirely forsake OXPHOS, as evidenced by studies indicating that, under specific conditions, certain cancer cells still depend on OXPHOS for energy production. Alterations in mitochondrial function and OXPHOS in cancer cells are intricately linked to factors such as tumor microenvironment acidification, escalated reactive oxygen species (ROS) production, and mitochondrial DNA mutations [45, 46]. These insights underscore the pivotal role of MRPL12 in the etiology and advancement of diverse pathological states. Reports have demonstrated that LUAD cells modulate tumor growth and dissemination by influencing OXPHOS efficiency and regulating the accrual of energy and metabolic intermediates [47, 48]. Our investigation revealed notable changes in mitochondrial structure, diminished OXPHOS, reduced ATP production, lowered mtDNA copy number, and impaired mitochondrial protein biosynthesis in MRPL12-deleted cells. Our findings suggest that MRPL12 influences LUAD progression by affecting mitochondrial homeostasis, particularly mitochondrial OXPHOS. Seahorse analysis revealed that MRPL12 knockdown primarily impacted mitochondrial OXPHOS but did not significantly affect glycolysis. Further investigation is needed to determine whether MRPL12 also regulates other metabolic pathways, such as fatty acid oxidation and glutamine metabolism. As a newly identified regulator of mitochondrial transcription, MRPL12 plays a crucial role in the regulation of mitochondrial energy metabolism. We hypothesize that MRPL12 may also promote cancer progression in other tumors, especially those primarily driven by mitochondrial oxidative phosphorylation.

To unravel MRPL12‘s potential mode of action and identify intervention targets in LUAD, we conducted protein mass spectrometry. UBASH3B, a member of the TULA family renowned for its potent protein tyrosine phosphatase activity, engages in diverse biological processes by interacting with multiple proteins [49]. In our study, different experiments verified that UBASH3B could specifically interact with MRPL12 (Fig. 5). We further found that UBASH3B, as a tyrosine phosphatase, can regulate the phosphorylation level of MRPL12, which is the first time to report the phosphorylation of MRPL12 and MRPL12 as a new substrate for UBASH3B. Mechanically, we found that UBASH3B can affect the binding of MRPL12 and POLRMT through its phosphatase function at 60th tyrosine thus participating in the transcriptional regulation of mitochondrial genes, and thus affecting the oxidative phosphorylation capacity of mitochondria. In this study, we also found that MRPL12 and UBASH3B also cause changes in mtDNA copy number, so there may be other regulatory mechanisms of MRPL12 in mitochondrial energy metabolism. Elevated UBASH3B expression has been documented in various cancers, fostering invasive and metastatic behavior and correlating with poor survival in breast and prostate cancer patients [39, 50]. However, we found that UBASH3B is downregulated in LUAD, and its low expression is significantly associated with poor prognosis. Moreover, overexpression of UBASH3B alleviated the proliferative and migratory phenotypes induced by MRPL12 overexpression. These findings suggest that UBASH3B may act as a critical inhibitor of MRPL12 function in LUAD development and progression. The literature and our study indicate that UBASH3B expression varies across different tumor types, potentially exerting opposite effects in different cancers.

Notably, we observed that UBASH3B binds to MRPL12 and significantly influences the tyrosine phosphorylation level of MRPL12 Y60. Phosphorylation, a crucial process in protein post-modification, plays a pivotal role in cellular signal transduction. In cancer, aberrant phosphorylation of specific proteins closely correlates with tumor occurrence, development, and metastasis [51]. Our study also reveals that the MRPL12 Y60A mutation (which mimics Y60 dephosphorylation) alters mitochondrial structure, reduces MRPL12 binding to POLRMT, and consequently diminishes mitochondrial transcription and oxidative phosphorylation capability. These changes impact the proliferation, migration, and invasion of LUAD cells. Additionally, we developed an antibody specific for MRPL12 Y60 phosphorylation and observed elevated levels of MRPL12 Y60 phosphorylation in LUAD tissues. For the first time, we identified the key phosphorylation site of MRPL12 and elucidated its impact on tumors in murine models and LUAD patients. These findings reinforce the proposition that MRPL12 Y60 phosphorylation is pivotal for mitochondrial metabolism and could serve as an intervention target for LUAD. Therefore, designing small molecule compounds targeting the MRPL12 Y60 site to inhibit MRPL12 phosphorylation represents a potential intervention strategy for LUAD. Furthermore, changes in phosphorylation levels at MRPL12 Y60 in other tumors and the role of this modification in tumor development have not been reported. Thus, it is important to explore whether MRPL12 Y60 phosphorylation has similar effects in other cancers, as this could provide a theoretical basis for targeting this site in various tumors. While the Warburg effect is a hallmark of tumor metabolism and a key energy source for tumor growth, our study highlights that enhanced mitochondrial OXPHOS is a distinctive feature of LUAD metabolism. Interfering with MRPL12 Y60 phosphorylation to disrupt mitochondrial metabolism could serve as a promising intervention strategy for LUAD.

However, our study presents several limitations. Firstly, our investigation revealed a significant correlation between heightened MRPL12 expression and both pathologic stages and TNM stages in LUAD. Nevertheless, we abstained from delving into the causal relationship between increased MRPL12 expression and advanced pathologic stages, or vice versa. Secondly, the potential utility of heightened MRPL12 expression as a marker for pathologic stages or TNM stage of LUAD warrants further elucidation through expanded clinical specimen analysis. Thirdly, while we confirmed the phosphorylation of the Y60 site as MRPL12 active site, we did not proceed to screen small molecular compounds as potential targeted drugs to impede LUAD progression.

In summary, our investigation has delineated the pivotal metabolic reprogramming role and molecular mechanism of MRPL12 in LUAD, employing LUAD patient-derived organoids, the *LSL-p53*^+*/*+^*;LSL-Kras*^*G12D*^ LUAD mouse model, as well as LUAD tissues and cells. Moreover, we identified MRPL12 Y60 phosphorylation as a key modification associated with the occurrence and development of LUAD, highlighting its potential as a therapeutic target for the disease.

## Materials and methods

### Mice

Transgenic LSL-Kras^G12D/+^ and LSL-p53^+/+^ mice were kindly provided by Pengju Zhang (Shandong university, China). MRPL12^flox/flox^ mice were generated by Shanghai Model Organisms Center, Inc (Shanghai, China). Mice were crossed and genotyped to obtain animals with the following genotype:LSL-p53^+/+^; LSL-Kras^G12D^;MRPL12^flox/flox^. Genotypes were determined by PCR. Only littermate mice were used in all experiments. All procedures were approved by the Ethics Review Committee of Shandong Provincial Hospital Affiliated to Shandong First Medical University.

#### Cell culture

The lung cancer cell lines (BEAS-2B, A549, H1288, H1975, H838, PC-9, HEK293T, and HUVEC) were purchased from the cell bank of Shanghai Institute of Biosciences (Shanghai, China). BEAS-2B, H1299, H838, PC-9, and HEK293T cell lines were cultured in RPMI1640 (Gibco®Grandisland, NY) containing 10% fetal bovine serum (FBS, Gibco®Grandisland, NY) and antibiotics (100 U/mL penicillin and 100 μg/mL streptomycin). A549 cells were cultured in F12K medium (HyClone) containing 10% FBS and antibiotics. HUVEC cells were cultured in a HUVEC specific complete medium (Procell). All cells were maintained in a humidified atmosphere at 37 °C containing 5% CO2.

### Patients and specimens

Human LUAD tissues and paired normal tissues were gained from LUAD patients after surgery at the Provincial Hospital of Shandong First Medical University. The human LUAD tissue microarray (Shanghai Outdo Biotech Co., Ltd., Shanghai, China) used in this study was constructed with specimens from 75 patients with LUAD who underwent curative resection. All the patients did not have adjuvant chemotherapy or radiotherapy. It was approved by the Ethical Committee of Shanghai Outdo Biotech Co.td.

### LUAD organoid

LUAD tissues for organoid culture were derived LUAD patients after surgery at the Shandong Provincial Hospital. After surgery, LUAD tissues werecut into small 1-3mm^3^ pieces using surgical scissors. Digestion of tissue fragments at 37 °C for 30 min using tumor tissue digestive solution (Biogenous, China). FBS was added to the tissue digest mixture to achieve a final concentration of 2% and filtered using a 100um cell strainer. Collect cells by centrifugation at 250 g for 3 min and discard the filtrate. Resuspension particles were used in tumor organoid basal medium (Biogenous, China). Centrifuge the suspension at 4 ℃ and 250 g for 3 min, repeating the procedure once more. The cells were collected by centrifugation, resuspended in BME (Corning), plated in 24-well plates, and expanded as 3D organoids. Put 24-well plates in a humidified incubator of 37 ℃ and 5% carbon dioxide for 40 min to allow the BME solidify. Add 500μL of organoid complete medium (Biogenous, China) to each well. Medium was changed every 4 days and PDO were passaged every 2 weeks. Incubate the culture plates in a humidified incubator at 37 ℃ and 5% CO^2^ for 7–10 days.

### Ad-Cre infection of mouse lung

Six-to eight-week-old mice were anesthetized with isoflurane via a gas chamber and were infected with intratracheal adenoviral-Cre (Ad-Cre; SyngenTech) at a dose of 5 × 10^^7^ PFU as previously described. At weeks 18 postinfection, the lungs were obtained.

### Silencing or overexpression of MRPL12

The lentiviral particles encoding the MRPL12 shRNA or the MRPL12 overexpressing construct were gained from Shanghai Genomeditech Co (Shanghai, China). The lentiviral particles encoding the MRPL12 Y60A overexpressing construct were gained from Beijing SyngenTech Co(Beijing China). LUAD cells were maintained at 40% confluence and infected with the lentiviral particles. Afterwards, medium was replaced with complete medium containing 1.5 μg/mL puromycin for four passages. Expressions of MRPL12 mRNA and protein in stable cells were verified by qRT-PCR and western blotting assays.

### Immunohistochemistry

For immunohistochemical staining, LUAD samples were fixed, paraffin-embedded, and sectioned into 3 μm thick slices. Tissue slides were prepared and deparaffinized by baking in oven 65 °C for 2 h,and then dewaxed by gradient ethanol. Goat serum was used for blocking. Slides were then stained with primary antibody overnight at 4℃ and secondary antibodies(Goat anti-rabbit antibody, Zhongshan Biotechnology Co, Beijing, China) were incubated for 1 h at room temperature. Finally, DAB staining, hematoxylin redyeing and hydrochloric acid alcohol differentiation. The images were captured with a Olympus microscope imaging system (Olympus, Tokyo, Japan) and were analyzed by Image J (version 1.52, NIH, USA). The following primary antibodies were used: MRPL12(1:250 dilution; Proteintech Cat# 14,795–1-AP), ND1 (1:100 dilution; Proteintech Cat# 19,703–1-AP), MTCO2 (1:300 dilution; Proteintech Cat# 55,070–1-AP), UBASH3B (1:1000 dilution; Proteintech Cat# 19,563–1-AP), Phospho-MRPL12 Y60(1:50 dilution; QiangYao), Ki67 (1:2000 dilution; Proteintech Cat# 27,309–1-AP), Cytokeratin 7 (1:1000; Proteintech Cat# 17,513–1-AP).

### IHC Score

Double-blind scoring was performed by Two experienced pathologists according to the following methodology (immunoreaction score, IRS): The intensity of cell staining was graded into 4 levels, with no positive staining (negative) scoring 0, yellowish (weakly positive) scoring 1, brown-yellow (positive) scoring 2, and brown (strongly positive) scoring 3. The percentage of positive cells was graded into 4 levels, with ≤ 25% scoring 1, 26%-50% scoring 2, 51%-75% scoring 3, and > 75% scoring 4, and the two scores were multiplied to arrive at the final scoring result.

### siRNAs and plasmids

Overexpression plasmids of MRPL12 and the control empty vector were purchased from Genomeditech (Shanghai, China). One plasmid with full-length UBASH3B (FL) and three plasmids with UBASH3B deletion mutants were constructed by BioSune (Shanghai, China). UBASH3B siRNA were synthesized by RiboBio (Guangzhou, CN). Cells in 6-well plates were transfected with plasmids using Lipofectamine 3000 (Invitrogen, Shanghai, China) or with siRNA using riboFECTTM CP Transfection kit (RiboBio, Guangzhou, China) according to the manufacturer’s instructions.

### MRPL12 KO

A CRISPR/Cas9-MRPL12-KO construct encoding the sgRNA specifically targeting MRPL12 (sgRNA2-CTCCTTGGGGGCGTTATCCA) was produced from Weizhen(Jinan China). sgRNA was cloned into lentiCrisprV2 plasmid, which was co-transfected with VSV-G plasmid and psPAX2 plasmid into HEK 293 T cells at the ratio of 500 ng:250 ng:250 ng. Forty-eight hours later, the supernatants containing packaged lentiviruses were collected and used to transduce LUAD cells and PDO which were then selected using puromycin (1.6 μg/mL). Control cells were transduced with the CRISPR/Cas9 control construct.

### Western blot

Cells were incubated with the RIPA lysis buffer (Solarbio Biotechnology, Beijing, China)containing protease and phosphatase inhibitor cocktail (Solarbio, Beijing, China). and the quantified protein lysates were separated on 8%-12% PAGE mini-gels. Then the protein was transferred into 0.45 µm PVDF membranes (Millipore, United States) by electroblot for 1 h Membranes were blocked in TBST, containing 5% no-fat milk for 1 h and then incubated in primary antibodies at 4℃ overnight. And then the membranes were incubated with an HRP-conjugated goat-anti rabbit (mouse) secondary antibody (Invitrogen, Shanghai, China) and detected by enhanced chemiluminescence reagents (ECL, Millipore, United States). The bands were visualized by ChemiScope 6000 (Clinx, Shanghai, China), and analyzed by Image J (version 1.52, NIH, USA). The following primary antibodies were used:MRPL12(1:2000 dilution; Proteintech Cat# 14,795–1-AP), ACTIN(1:5000 dilution; Proteintech Cat# 81,115–1-RR), ND1(1:1000 dilution; Proteintech Cat# 19,703–1-AP), CYTB(1:1500 dilution; Proteintech Cat# 55,090–1-AP), MTCO2(1:3000 dilution; Proteintech Cat# 55,070–1-AP), UBASH3B(1:1000 dilution; Proteintech Cat# 19,563–1-AP), POLRMT(1:1500 dilution; Abclonal Cat# A15605), MRPL11(1:3000 dilution; Proteintech Cat# 15,543–1-AP), Pan Phospho-Tyrosine(1:1000 dilution; Abclonal Cat# AP0905; AP1162), Phospho-MRPL12 Y60(1:500 dilution; ChinaPeptides).

### Real-time polymerase chain reaction (RT-PCR)

Total RNA was extracted using TRIzol Reagent (Invitrogen, United States) as manufacturer’s instructions described and first strand cDNA synthesis was performed using Revert Aid First Strand cDNA Synthesis Kit (Thermo scientific). Real-time quantitative polymerase chain reaction (RT-qPCR) was conducted using SYBR Green qPCR Master Mix (ACCURATE BIOTECHNOLOGY, Changsha, China) on a Roche 480II system as follows: initially, 95 °C for 2 min for denaturation, followed by 40 cycles at 95 °C for 15 s and 60 °C for 1 min, then 95 °C for melting, and finally 50 °C for 30 s for cooling. The primers sequences were as following:

- β-actin: F-TGGCACCCAGCACAATGAA;
- R-CTAAGTCATAGTCCGCCTAGAAGCA;
- MRPL12: F-ATCCAGGATGTCGGGCTTG;
- R-TGATGCCTTGGATGTAGTTCTTGA;
- ND1:F-CGAGCAGTAGCCCAAACAATC;
- R-GATGGCAGGAGTAATCAGAGGTG;
- CYTB:F-CCCACCCCATCCAACATCTC; R-GCGTCTGGTGAGTAGTGCAT;
- MTCO2:F-CATGAGCTGTCCCCACATTAG; R-CGGTCGTGTAGCGGTGAAA;
- UBASH3B:F-TGGTTTCCGAGATTACGAGAAA;
- R-TTTTGTCCACTCAAATAAGCCG.

### Mitochondrial DNA copy number Quantification

Total DNA was harvested from treated cells using FastPure Cell/Tissue DNA Isolation Mini Kit (Vazyme, DC102-01). To measure mtDNA content, we used 1 μg DNA and primers to the Journal Pre-prooD-loop region of the mitochondrial genome. G6PC primers served as the nuclear control to normalize the mitochondrial to nuclear gene ratio.The primers sequences were as following:

- D-Loop 2: F-GGCTCTCAACTCCAGCATGT; R- AGGACGAGGGAGGCTACAAT;
- G6PC: F-CTGTCTTTGATTCCTGCCTCAT; R- GTGGCTGTGCAGACATTCAA.

### Immunofluorescence staining

Cells seeded on chamber slides were treated for 24 h. For mitochondrial staining, cells were incubated with 100 nM MitoTracker Red CMXRos (Molecular Probes) at 37 °C for 30 min. Then, cells were fixed with 4% paraformaldehyde. After washing 3 times for 5 min with PBS, cells were permeabilized in 0.1% Triton X-100 and blocked for 1 h with 1% goat serum (Solarbio, SL038), and incubated with primary antibody overnight at 4 °C. This was followed by 1 h incubation at 37 °C with secondary antibody. After incubation, nuclei were counterstained with Hoechst 33,342 (Life technologies, H3570). Fluorescent images were taken with Olympus microscope imaging system (Olympus, Tokyo, Japan) and analyzed by Image J. The following primary antibodies were used: MRPL12 (1:200 dilution; Proteintech Cat# 14,795–1-AP), ND1 (1:100 dilution; Proteintech Cat# 19,703–1-AP), CYTB (1:150 dilution; Proteintech Cat# 55,090–1-AP), MTCO2 (1:300 dilution; Proteintech Cat# 55,070–1-AP), UBASH3B (1:100 dilution; Proteintech Cat# 19,563–1-AP).

### SIM image acquisition and reconstruction

The SIM images were performed using a commercial Zeiss microscope (ELYRA 7 with Lattice-SIM, Carl Zeiss Microimaging). A549 cells were seeded in a 20-mm Glass Bottom Cell Culture Dish (NEST) and incubated with 100 nM MitoTracker Red CMXRos in F12K medium at 37 °C for 30 min. SIM processing was performed using the SIM module in the Zen black software package (Carl Zeiss Microimaging).

### Tumor growth in vivo

Four-to-five-week-old BALB/c nude mice were utilized in this study. For xenograft assay, mice were subcutaneously injected with A549 cells that underwent different treatments(5 × 10^6 cells in 100 μL of PBS and Matrigel per mouse). Tumor size was measured on a weekly basis, and tumor volume was calculated using the formula: volume (mm^3^) = tumor length × width2/2. Within 4 weeks xenograft-bearing nude mice were established and the volume of each xenograft was close to 100 mm^3^. After four weeks, all mice were sacrificed, and tumor volume and weight were compared. Tumors were fixed in 10% formalin and embedded in paraffin for subsequent IHC examination.

To examine lung cancer cell metastasis, A549 cells, stably expressing luciferase and lentivirus, were injected into the tail vein of BALB/c nude mice (1 × 10^7 mL, 0.1 mL per mouse). After 36 days, 3 mice from each group were randomly selected for bioluminescent analysis. Bioluminescent signal was induced by intraperitoneal injection with 150 mg/kg D-luciferin (Meilunbio, MB1834) and imaged by the IVIS Lumina III Spectrum System (Perkin-Elmer, Waltham, MA, USA) after 10 min.

### In situ proximity ligation assay

A549 cells were fixed in 4% paraformaldehyde and permeabilized in 0.5% Triton X-100. The PLA assay was carried out according to manufacturer’s instructions (DUO92101, Sigma) with rabbit anti-UBASH3B antibody (19,563–1-AP, Proteintech, 1:100) and mouse anti-MRPL12 antibody (sc-100839, Santa, 1:50). The signal was visualized using an IXplore SpinSR10 (Olympus, Tokyo, Japan).

### Immunoprecipitation assay

Immunoprecipitation assay (IP) was carried out using Protein A/G Magnetic Beads.(HY-K0202, MCE) according to the manufacturer’s instructions. Cells were lysed with IP Lysis Buffer. Lysates (1 mg) were incubated at 4 °C overnight with 4 mg (rabbit anti-MRPL12 antibody or rabbit control IgG antibody) and Protein A/G Beads on a rotator. The eluent was analyzed by western blotting.

### Mass spectrometry-based proteomics

A549 cells were lysed in Pierce IP Lysis Buffer containing protease and phosphatase inhibitor cocktail. Lysates were incubated overnight with anti-MRPL12 rabbit polyclonal antibody crosslinked Protein A/G Magnetic Beads. The beads were washed three times with washing buffer (PBST), and the proteins were heated to 95 ℃ in 1X loading buffer. Samples were separated on a 12% SDS‒PAGE gel. After gel staining with Coomassie bright blue (PA101-01, TIANGEN, Beijing), the gel was cut into several small bands, and the gel fragments were sent for protein identification by liquid chromatography with tandem mass spectrometry (LC‒MS/MS).

### Soft agar assay

First, 1 ml of 1.2% agarose in 2X RPMI-1640 medium containing 20% FBS was poured into each well of a 6-well plate and allowed to solidify at room temperature. Then, 2000 cells were suspended in 1 ml of 0.7% agarose in the same medium and plated on top of the base layer (three wells per group). The cells were cultured for 14 days, and the resulting colonies were observed and quantified under a microscope.

### Transwell assays

Transwell assays were performed with BD chambers (8-mm pores; BD Biosciences, Shanghai, China). About 1 × 10^5 cells/well were seeded in the upper chamber and cultured in serum-free medium. Medium with 30% serum was placed in the lower chambers. After migration through the Transwell membrane, the cells were fixed with 4% paraformaldehyde and stained with crystal violet (Solarbio, Beijing, China). The difference between the migration and invasion assays was the Transwell chambers for migration assays were not coated with Matrigel.

### EdU staining

LUAD cells with applied genetic modifications were seeded in 96-well plates at a density of 8 × 10^3^ cells per well and were cultured for 24 h. Afterwards, cells were fixed and permeabilized with 1% formaldehyde and 0.3% Triton X-100, respectively. EdU and DAPI fluorescence dyes were added, and cell nuclei were visualized under the EVOS M7000 (ThermoFisher, USA). EdU-positive nuclei ratio (% vs. DAPI) was recorded.

### Statistical analysis

The data are presented in bar plots as mean ± standard deviation (SD) of at least three independent experiments. The Shapiro–Wilk test was used to assess the normality of the data, and the F-test is used to assess the homogeneity of the data. For comparisons between two or more groups of quantitative data, we used Student’s t-test and analysis of variance (ANOVA), respectively. Welcht’s test was used to analyze normally distributed data with unequal variances. Non-normally distributed data were analyzed using Mann–Whitney test. Kaplan–Meier analysis was used for survival analysis, and the statistical significance of prognosis between different groups was compared using log-rank test. A *p*-value of less than 0.05 (two-tailed) was considered statistically significant. All statistical analyses were conducted using Prism 8.0 (GraphPad Software, San Diego, CA). Graph abstract was drawn by Figdraw.

## Supplementary Information

**Supplementary Material 1: Figure S1.** MRPL12 is highly expressed in LUAD organoid, tissues, and cells and associated with poor survival. A Analysis of MRPL12 expression in KRAS mutant versus non-mutant LUAD tissues using TCGA databases. B Analysis of the correlation between MRPL12 and TP53 expression in LUAD tissues using TCGA databases. C Organoid morphology. Scale bar: 50 μm. D MRPL12 mRNA levels in LUAD. E MRPL12 mRNA levels in deceased and living LUAD patients. F Analysis of MRPL12 expression levels based on T, N, M clinical stage, and pathologic stage. G Overall survival analysis based on MRPL12 mRNA levels in LUAD. H Kaplan-Meier survival curves depicting the correlation between MRPL12 mRNA levels and the overall survival time of patients with lung squamous cell carcinoma. I-J Western blot and RT-PCR were employed to assess the efficiency of MRPL12 knockdown or overexpression in the indicated cells (I) and PDOs (J). **, *p*<0.01;***, *p*<0.001; ****, *p*<0.0001. **Figure S2.** MRPL12 facilitates LUAD tumorigenesis in vitro. A-B Soft agar (A) and EDU (B) assays were conducted to evaluate cell proliferation and colony formation capabilities in A549 and H1299 cells with stable MRPL12 overexpression or knockdown. C EDU assays were performed to assess cell proliferation in A549 and H1299 cells with MRPL12 knockout or re-expression. D Transwell assays were conducted to assess cell migration and invasion ability in A549 and H1299 cells with stable MRPL12 overexpression or knockdown. E Transwell assays were conducted to assess cell migration and invasion ability in A549 and H1299 cells with MRPL12 knockout or re-expression. F Quantification of A549 and H1299 cells in trans-endothelial migration assays (referenced in Figure 3H). G-H Ingenuity Pathway Analysis (IPA) of the differentially expressed genes in A549 cells with MRPL12 knockdown. *, *p*<0.05; **, *p*<0.01; ***, *p*<0.001; ****, *p*<0.0001. **Figure S3.** Co‐expression analysis combined with KEGG enrichment analysis. A Co‐expression analysis of MRPL12. B Western blotting was used to analyze the expression of the indicated genes following MRPL12 knockdown or overexpression in A549 and H1299 cells. *, *p*<0.05; **, *p*<0.01; ***, *p*<0.001, ****, *p*<0.0001; ns, *p*>0.05. **Figure S4.** MRPL12 promotes lung tumorigenesis via promoting mitochondrial oxidative phosphorylation. A Analysis of mtDNA copy number in H1299 cells subjected to MRPL12 knockdown or overexpression. B-C Evaluation of mRNA (B) and protein (C) levels of OXPHOS complexes in H1299 cells following MRPL12 knockdown or overexpression. D Measurement of OCR in H1299 cells with MRPL12 knockdown or overexpression using Seahorse XFe96. E Analysis of mitochondrial OXPHOS, including basal respiration, maximal respiration, ATP production, and spare respiratory capacity in H1299 cells with MRPL12 knockdown or overexpression. F Mitochondrial mass was measured using MitoTracker Red CMXRos. G-H Examination of the proliferation and migration capabilities of A549 and H1299 cells overexpressing MRPL12 under treatment with an OXPHOS inhibitor. *, *p*<0.05; **, *p*<0.01;***, *p*<0.001; ****, *p*<0.0001; ns, *p*>0.05. **Figure S5.** UBASH3B interacts with MRPL12. A Analysis of UBASH3B expression across various cancer types using pan-cancer samples from the TIMER2.0 database. B Examination of UBASH3B expression in LUAD and corresponding normal tissues within the TCGA databases. C Assessment of UBASH3B expression in LUAD and adjacent normal tissues through analysis of the TCGA databases. D Comparative analysis of UBASH3B expression in LUAD and normal tissues utilizing the GEPIA2 website. E-F Kaplan-Meier survival curves illustrating the impact of UBASH3B expression on the overall survival of LUAD patients. G-H Evaluation of the migration (G) and proliferation (H) capabilities of A549 and H1299 cells with UBASH3B overexpression or knockdown. I-J Investigation of the proliferation and migration properties of A549 and H1299 cells overexpressing MRPL12, along with UBASH3B H391A or UBASH3B WT overexpression, assessed by EDU and Transwell assays. *, *p*<0.05;**, *p*<0.01; ***, *p*<0.001; ****, *p*<0.0001. **Figure S6.** The expression and role of UBASH3B in LUAD. A UBASH3B expression was assessed in LUAD tissues and PDO. B-C Analysis of the correlation between UBASH3B expression levels and TNM stages or pathological stage using TCGA datasets. D Transwell assays were conducted to assess cell migration and invasion ability in A549 and H1299 cells with UBASH3B knockdown, combined with MRPL12 WT, MRPL12 Y60A or MRPL12 Y60E overexpression. E EDU assays were conducted to assess cell proliferation in A549 and H1299 cells with UBASH3B overexpression, combined with MRPL12 WT, MRPL12 Y60E or MRPL12 Y60A overexpression. *,*p*<0.05; **, *p*<0.01; ***, *p*<0.001; ****, *p*<0.0001. **Figure S7.** UBASH3B dephosphorylates MRPL12 at residue Y60 and inhibits lung tumorigenesis. A-B Assessment of protein (A) and mRNA (B) levels of OXPHOS complexes in H1299 cells overexpressing the MRPL12 Y60A mutation or MRPL12 WT. C Analysis of mtDNA copy number in H1299 cells with overexpression of MRPL12 WT or the MRPL12 Y60A mutant. D Examination of oxygen consumption rate (OCR) in H1299 cells overexpressing MRPL12 Y60A or MRPL12 WT, performed using Seahorse XFe96. E Quantification of basal respiration, ATP production, maximal respiration, and spare respiratory capacity in H1299 cells overexpressing MRPL12 Y60A or MRPL12 WT. F Co-ip assays conducted in A549 and H1299 cells, wherein cells were lysed and immunoprecipitated with an MRPL12 antibody, followed by western blot analysis with POLRMT. G-H Analysis of proliferation and migration characteristics in A549 and H1299 cells with overexpression of MRPL12 WT, MRPL12 Y60A, or Y152A mutations. *, *p*<0.05; **, *p*<0.01; ***,*p*<0.001; ****, *p*<0.0001; ns, *p*>0.05.

## Data Availability

All the data that support the findings of this study are available from the corresponding author upon reasonable request.

## Abbreviations

LUAD: Lung adenocarcinoma
PDO: Patient-derived organoids
NSCLC: Non-small cell lung cancer
OXPHOS: Oxidative phosphorylation
MRPL12: Mitochondrial ribosomal protein L7/L12
ROS: Reactive oxygen species
PTM: Posttranslational modifications
UBASH3B: Ubiquitin Associated and SH3 Domain Containing B
KP: *Tp53* ^*fl/fl*^ *;Kras* ^*G12D*^
KPM-HET: *Tp53* ^*fl/fl*^ *;Kras* ^*G12D*^ *;MRPL12* ^*fl/*^^+^
KPM: *Tp53* ^*fl/fl*^ *;Kras* ^*G12D*^ *;MRPL12* ^*fl/fl*^
Ad-Cre: Adenoviral delivery of Cre recombinase
HE: Hematoxylin–eosin
IHC: Immunohistochemical
TEM: Trans-microvascular endothelial migration
IPA: Ingenuity Pathway Analysis
SIM: Structured illumination super-resolution microscopy
MS: Mass spectrometry
co-IP: Co-immunoprecipitation
IF: Immunofluorescence
POLRMT: RNA polymerase

## References

[1] Sung H, Ferlay J, Siegel RL, Laversanne M, Soerjomataram I, Jemal A, Bray F. Global Cancer Statistics 2020: GLOBOCAN Estimates of Incidence and Mortality Worldwide for 36 Cancers in 185 Countries. CA Cancer J Clin. 2021;71(3):209–49. 33538338 10.3322/caac.21660

[2] Ma L, Xue X, Zhang X, Yu K, Xu X, Tian X, Miao Y, Meng F, Liu X, Guo S, et al. The essential roles of m(6)A RNA modification to stimulate ENO1-dependent glycolysis and tumorigenesis in lung adenocarcinoma. J Exp Clin Cancer Res. 2022;41(1):36. 35078505 10.1186/s13046-021-02200-5 PMC8788079

[3] Herbst RS, Morgensztern D, Boshoff C. The biology and management of non-small cell lung cancer. Nature. 2018;553(7689):446–54. 29364287 10.1038/nature25183

[4] Howington JA, Blum MG, Chang AC, Balekian AA, Murthy SC. Treatment of stage I and II non-small cell lung cancer: Diagnosis and management of lung cancer, 3rd ed: American College of Chest Physicians evidence-based clinical practice guidelines. Chest. 2013;143(5 Suppl):e278S–e313S. 23649443 10.1378/chest.12-2359

[5] Calvayrac O, Pradines A, Pons E, Mazières J, Guibert N. Molecular biomarkers for lung adenocarcinoma. Eur Respir J. 2017;49(4):1601734. 10.1183/13993003.01734-2016 28381431

[6] Hanahan D, Weinberg Robert A. Hallmarks of Cancer: The Next Generation. Cell. 2011;144(5):646–74. 21376230 10.1016/j.cell.2011.02.013

[7] Sainero-Alcolado L, Liaño-Pons J, Ruiz-Pérez MV, Arsenian-Henriksson M. Targeting mitochondrial metabolism for precision medicine in cancer. Cell Death Differ. 2022;29(7):1304–17. 35831624 10.1038/s41418-022-01022-y PMC9287557

[8] Lin S, Huang C, Gunda V, Sun J, Chellappan SP, Li Z, Izumi V, Fang B, Koomen J, Singh PK, et al. Fascin Controls Metastatic Colonization and Mitochondrial Oxidative Phosphorylation by Remodeling Mitochondrial Actin Filaments. Cell Rep. 2019;28(11):2824–2836.e2828. 31509745 10.1016/j.celrep.2019.08.011 PMC6759858

[9] Rao S, Mondragón L, Pranjic B, Hanada T, Stoll G, Köcher T, Zhang P, Jais A, Lercher A, Bergthaler A, et al. AIF-regulated oxidative phosphorylation supports lung cancer development. Cell Res. 2019;29(7):579–91. 31133695 10.1038/s41422-019-0181-4 PMC6796841

[10] Wu C, Liu Ye, Liu W, Zou T, Lu S, Zhu C, He L, Chen J, Fang L, Zou L, et al. NNMT‐DNMT1 Axis is Essential for Maintaining Cancer Cell Sensitivity to Oxidative Phosphorylation Inhibition. Adv Sci. 2023;10(1):2202642. 10.1002/advs.202202642 PMC9811437 36382559

[11] Han M, Bushong EA, Segawa M, Tiard A, Wong A, Brady MR, Momcilovic M, Wolf DM, Zhang R, Petcherski A, et al. Spatial mapping of mitochondrial networks and bioenergetics in lung cancer. Nature. 2023;615(7953):712–9. 36922590 10.1038/s41586-023-05793-3 PMC10033418

[12] Guilbaud E, Barouillet T, Ilie M, Borowczyk C, Ivanov S, Sarrazy V, Vaillant N, Ayrault M, Castiglione A, Rignol G, et al. Cholesterol efflux pathways hinder KRAS-driven lung tumor progenitor cell expansion. Cell Stem Cell. 2023;30(6):800–817.e809. 37267915 10.1016/j.stem.2023.05.005

[13] Xu J-Y, Zhang C, Wang X, Zhai L, Ma Y, Mao Y, Qian K, Sun C, Liu Z, Jiang S, et al. Integrative Proteomic Characterization of Human Lung Adenocarcinoma. Cell. 2020;182(1):245–261.e217. 32649877 10.1016/j.cell.2020.05.043

[14] Ashton TM, McKenna WG, Kunz-Schughart LA, Higgins GS. Oxidative Phosphorylation as an Emerging Target in Cancer Therapy. Clin Cancer Res. 2018;24(11):2482–90. 29420223 10.1158/1078-0432.CCR-17-3070

[15] Surovtseva YV, Shutt TE, Cotney J, Cimen H, Chen SY, Koc EC, Shadel GS. Mitochondrial Ribosomal Protein L12 selectively associates with human mitochondrial RNA polymerase to activate transcription. Proc Natl Acad Sci. 2011;108(44):17921–6. 22003127 10.1073/pnas.1108852108 PMC3207702

[16] Cheong A, Lingutla R, Mager J. Expression analysis of mammalian mitochondrial ribosomal protein genes. Gene Expr Patterns. 2020;38: 119147. 32987154 10.1016/j.gep.2020.119147 PMC7726062

[17] Nouws J, Goswami AV, Bestwick M, McCann BJ, Surovtseva YV, Shadel GS. Mitochondrial Ribosomal Protein L12 Is Required for POLRMT Stability and Exists as Two Forms Generated by Alternative Proteolysis during Import. J Biol Chem. 2016;291(2):989–97. 26586915 10.1074/jbc.M115.689299 PMC4705416

[18] Gu X, Liu Y, Wang N, Zhen J, Zhang B, Hou S, Cui Z, Wan Q, Feng H. Transcription of MRPL12 regulated by Nrf2 contributes to the mitochondrial dysfunction in diabetic kidney disease. Free Radical Biol Med. 2021;164:329–40. 33444714 10.1016/j.freeradbiomed.2021.01.004

[19] Ji X, Chu L, Su D, Sun S, Wang Y, Mu Q, Liu Y, Wan Q. MRPL12-ANT3 interaction involves in acute kidney injury via regulating MPTP of tubular epithelial cells. iScience. 2023;26(5):106656. 37182101 10.1016/j.isci.2023.106656 PMC10173734

[20] Ji X, Yang X, Gu X, Chu L, Sun S, Sun J, Song P, Mu Q, Wang Y, Sun X et al. CUL3 induces mitochondrial dysfunction via MRPL12 ubiquitination in renal tubular epithelial cells. FEBS J. 2023;290(22):5340–52. 10.1111/febs.16919 37526061

[21] Wu X, Xu M, Geng M, Chen S, Little PJ, Xu S, Weng J. Targeting protein modifications in metabolic diseases: molecular mechanisms and targeted therapies. Signal Transduct Target Ther. 2023;8(1):220. 37244925 10.1038/s41392-023-01439-y PMC10224996

[22] Deribe YL, Pawson T, Dikic I. Post-translational modifications in signal integration. Nat Struct Mol Biol. 2010;17(6):666–72. 20495563 10.1038/nsmb.1842

[23] Han T, Wang Y, Cheng M, Hu Q, Wan X, Huang M, Liu Y, Xun W, Xu J, Wang L et al. Phosphorylated SHMT2 Regulates Oncogenesis Through m(6) A Modification in Lung Adenocarcinoma. Adv Sci (Weinh). 2024;11(18):e2307834. 10.1002/advs.202307834 PMC11095143 38460155

[24] Pan S, Chen R. Pathological implication of protein post-translational modifications in cancer. Mol Aspects Med. 2022;86:101097. 35400524 10.1016/j.mam.2022.101097 PMC9378605

[25] Wang H, Yang L, Liu M, Luo J. Protein post-translational modifications in the regulation of cancer hallmarks. Cancer Gene Ther. 2023;30(4):529–47. 35393571 10.1038/s41417-022-00464-3

[26] Singh V, Ram M, Kumar R, Prasad R, Roy BK, Singh KK. Phosphorylation: Implications in Cancer. Protein J. 2017;36(1):1–6. 28108801 10.1007/s10930-017-9696-z

[27] Skoulidis F, Heymach JV. Co-occurring genomic alterations in non-small-cell lung cancer biology and therapy. Nat Rev Cancer. 2019;19(9):495–509. 31406302 10.1038/s41568-019-0179-8 PMC7043073

[28] de Seranno S, Meuwissen R. Progress and applications of mouse models for human lung cancer. Eur Respir J. 2010;35(2):426–43. 20123848 10.1183/09031936.00124709

[29] Tong L, Shen S, Huang Q, Fu J, Wang T, Pan L, Zhang P, Chen G, Huang T, Li K, et al. Proteasome-dependent degradation of Smad7 is critical for lung cancer metastasis. Cell Death Differ. 2020;27(6):1795–806. 31767934 10.1038/s41418-019-0459-6 PMC7244558

[30] Zhang S, You X, Zheng Y, Shen Y, Xiong X, Sun Y. The UBE2C/CDH1/DEPTOR axis is an oncogene and tumor suppressor cascade in lung cancer cells. J Clin Invest. 2023;133(4):e162434. 10.1172/JCI162434 PMC9927933 36548081

[31] Kong Y, Allison DB, Zhang Q, He D, Li Y, Mao F, Li C, Li Z, Zhang Y, Wang J, et al. The kinase PLK1 promotes the development of Kras/Tp53-mutant lung adenocarcinoma through transcriptional activation of the receptor RET. Sci Signal. 2022;15(754):eabj4009. 36194647 10.1126/scisignal.abj4009 PMC9737055

[32] Kim M, Mun H, Sung CO, Cho EJ, Jeon H-J, Chun S-M, Jung DJ, Shin TH, Jeong GS, Kim DK, et al. Patient-derived lung cancer organoids as in vitro cancer models for therapeutic screening. Nat Commun. 2019;10(1):3991. 31488816 10.1038/s41467-019-11867-6 PMC6728380

[33] Liu Y, Zhou Y, Chen P. Lung cancer organoids: models for preclinical research and precision medicine. Front Oncol. 2023;13:1293441. 37941550 10.3389/fonc.2023.1293441 PMC10628480

[34] Gkatzis K, Taghizadeh S, Huh D, Stainier DYR, Bellusci S. Use of three-dimensional organoids and lung-on-a-chip methods to study lung development, regeneration and disease. Eur Respir J. 2018;52(5):1800876. 10.1183/13993003.00876-2018 30262579

[35] Xiao ZJ, Wang SQ, Chen JJ, Li Y, Jiang Y, Tin VP, Liu J, Hu H, Wong MP, Pan Y et al. The Dual Role of the NFATc2/galectin-9 Axis in Modulating Tumor-Initiating Cell Phenotypes and Immune Suppression in Lung Adenocarcinoma. Adv Sci (Weinh). 2024;11(20):e2306059. 10.1002/advs.202306059 PMC11132051 38528665

[36] Reymond N, d’Água BB, Ridley AJ. Crossing the endothelial barrier during metastasis. Nat Rev Cancer. 2013;13(12):858–70. 24263189 10.1038/nrc3628

[37] Liu J, Li C, Wang J, Xu D, Wang H, Wang T, Li L, Li H, Nan P, Zhang J, et al. Chromatin modifier MTA1 regulates mitotic transition and tumorigenesis by orchestrating mitotic mRNA processing. Nat Commun. 2020;11(1):4455. 32901005 10.1038/s41467-020-18259-1 PMC7479136

[38] Tsygankov AY. TULA-family proteins: Jacks of many trades and then some. J Cell Physiol. 2018;234(1):274–88. 30076707 10.1002/jcp.26890

[39] Lee ST, Feng M, Wei Y, Li Z, Qiao Y, Guan P, Jiang X, Wong CH, Huynh K, Wang J, et al. Protein tyrosine phosphatase *UBASH3B* is overexpressed in triple-negative breast cancer and promotes invasion and metastasis. Proc Natl Acad Sci. 2013;110(27):11121–6. 23784775 10.1073/pnas.1300873110 PMC3704014

[40] Pawlicki JM, Cookmeyer DL, Maseda D, Everett JK, Wei F, Kong H, Zhang Q, Wang HY, Tobias JW, Walter DM, et al. NPM-ALK-Induced Reprogramming of Mature TCR-Stimulated T Cells Results in Dedifferentiation and Malignant Transformation. Cancer Res. 2021;81(12):3241–54. 33619116 10.1158/0008-5472.CAN-20-2297 PMC8260452

[41] Jackson EL, Olive KP, Tuveson DA, Bronson R, Crowley D, Brown M, Jacks T. The Differential Effects of Mutant *p53* Alleles on Advanced Murine Lung Cancer. Can Res. 2005;65(22):10280–8. 10.1158/0008-5472.CAN-05-2193 16288016

[42] Chen Y-W, Huang SX, De Carvalho ALRT, Ho S-H, Islam MN, Volpi S, Notarangelo LD, Ciancanelli M, Casanova J-L, Bhattacharya J, et al. A three-dimensional model of human lung development and disease from pluripotent stem cells. Nat Cell Biol. 2017;19(5):542–9. 28436965 10.1038/ncb3510 PMC5777163

[43] Ji X, Yang Z, Li C, Zhu S, Zhang Y, Xue F, Sun S, Fu T, Ding C, Liu Y, et al. Mitochondrial ribosomal protein L12 potentiates hepatocellular carcinoma by regulating mitochondrial biogenesis and metabolic reprogramming. Metabolism. 2024;152:155761. 38104924 10.1016/j.metabol.2023.155761

[44] Warburg O. On the Origin of Cancer Cells. Science. 1956;123(3191):309–14. 10.1126/science.123.3191.309 13298683

[45] Vander Heiden MG, Cantley LC, Thompson CB. Understanding the Warburg Effect: The metabolic requirements of cell proliferation. Science. 2009;324(5930):1029–33. 19460998 10.1126/science.1160809 PMC2849637

[46] Wallace DC. Mitochondria and cancer. Nat Rev Cancer. 2012;12(10):685–98. 23001348 10.1038/nrc3365 PMC4371788

[47] Chuang C-H, Dorsch M, Dujardin P, Silas S, Ueffing K, Hölken JM, Yang D, Winslow MM, Grüner BM. Altered Mitochondria Functionality Defines a Metastatic Cell State in Lung Cancer and Creates an Exploitable Vulnerability. Can Res. 2021;81(3):567–79. 10.1158/0008-5472.CAN-20-1865 PMC8137518 33239425

[48] Kalainayakan SP, FitzGerald KE, Konduri PC, Vidal C, Zhang L. Essential roles of mitochondrial and heme function in lung cancer bioenergetics and tumorigenesis. Cell Biosci. 2018;8(1):56. 30410721 10.1186/s13578-018-0257-8 PMC6215344

[49] Tsygankov AY. TULA-family proteins: Jacks of many trades and then some. J Cell Physiol. 2019;234(1):274–88. 10.1002/jcp.26890 30076707

[50] Wang Z, Wang Y, Peng M, Yi L. UBASH3B Is a Novel Prognostic Biomarker and Correlated With Immune Infiltrates in Prostate Cancer. Front Oncol. 2020;9:1517. 32010618 10.3389/fonc.2019.01517 PMC6974685

[51] Ardito F, Giuliani M, Perrone D, Troiano G, Muzio LL. The crucial role of protein phosphorylation in cell signaling and its use as targeted therapy (Review). Int J Mol Med. 2017;40(2):271–80. 28656226 10.3892/ijmm.2017.3036 PMC5500920

