# Supplementary material for: UBASH3B-mediated MRPL12 Y60 dephosphorylation inhibits LUAD development by driving mitochondrial metabolism reprogramming
Source: J Exp Clin Cancer Res. 2024 Sep 30;43:268. doi: 10.1186/s13046-024-03181-x (PMC11441236; doi:10.1186/s13046-024-03181-x)
Supplement: Supplementary file 1 — Supplementary Material 1: Figure S1. MRPL12 is highly expressed in LUAD organoid, tissues, and cells and associated with poor survival. A Analysis of MRPL12 expression in KRAS mutant versus non-mutant LUAD tissues using TCGA databases. B Analysis of the correlation between MRPL12 and TP53 expression in LUAD tissues using TCGA databases. C Organoid morphology. Scale bar: 50 μm. D MRPL12 mRNA levels in LUAD. E MRPL12 mRNA levels in deceased and living LUAD patients. F Analysis of MRPL12 expression levels based on T, N, M clinical stage, and pathologic stage. G Overall survival analysis based on MRPL12 mRNA levels in LUAD. H Kaplan-Meier survival curves depicting the correlation between MRPL12 mRNA levels and the overall survival time of patients with lung squamous cell carcinoma. I-J Western blot and RT-PCR were employed to assess the efficiency of MRPL12 knockdown or overexpression in the indicated cells (I) and PDOs (J). **, p<0.01;***, p<0.001; ****, p<0.0001. Figure S2. MRPL12 facilitates LUAD tumorigenesis in vitro. A-B Soft agar (A) and EDU (B) assays were conducted to evaluate cell proliferation and colony formation capabilities in A549 and H1299 cells with stable MRPL12 overexpression or knockdown. C EDU assays were performed to assess cell proliferation in A549 and H1299 cells with MRPL12 knockout or re-expression. D Transwell assays were conducted to assess cell migration and invasion ability in A549 and H1299 cells with stable MRPL12 overexpression or knockdown. E Transwell assays were conducted to assess cell migration and invasion ability in A549 and H1299 cells with MRPL12 knockout or re-expression. F Quantification of A549 and H1299 cells in trans-endothelial migration assays (referenced in Figure 3H). G-H Ingenuity Pathway Analysis (IPA) of the differentially expressed genes in A549 cells with MRPL12 knockdown. *, p<0.05; **, p<0.01; ***, p<0.001; ****, p<0.0001. Figure S3. Co‐expression analysis combined with KEGG enrichment analysis. A Co‐expression [file 13046_2024_3181_MOESM1_ESM.zip › 13046_2024_3181_MOESM1_ESM/Supplementary Legends.docx]

**Figure S1. MRPL12 is highly expressed in LUAD organoid, tissues, and cells and associated with poor survival**

**A** Analysis of MRPL12 expression in KRAS mutant versus non-mutant LUAD tissues using TCGA databases. **B** Analysis of the correlation between MRPL12 and TP53 expression in LUAD tissues using TCGA databases. **C** Organoid morphology. Scale bar: 50 μm. **D** MRPL12 mRNA levels in LUAD. **E** MRPL12 mRNA levels in deceased and living LUAD patients. **F** Analysis of MRPL12 expression levels based on T, N, M clinical stage, and pathologic stage. **G**  Overall survival analysis based on MRPL12 mRNA levels in LUAD. **H**  Kaplan-Meier survival curves depicting the correlation between MRPL12 mRNA levels and the overall survival time of patients with lung squamous cell carcinoma. **I-J** Western blot and RT-PCR were employed to assess the efficiency of MRPL12 knockdown or overexpression in the indicated cells (I) and PDOs (J). **, p<0.01; ***, p<0.001; ****, p<0.0001;

**Figure S2. MRPL12 facilitates LUAD tumorigenesis in vitro**

**A-B** Soft agar (A) and EDU (B) assays were conducted to evaluate cell proliferation and colony formation capabilities in A549 and H1299 cells with stable MRPL12 overexpression or knockdown. **C** EDU assays were performed to assess cell proliferation in A549 and H1299 cells with MRPL12 knockout or re-expression. **D** Transwell assays were conducted to assess cell migration and invasion ability in A549 and H1299 cells with stable MRPL12 overexpression or knockdown. **E** Transwell assays were conducted to assess cell migration and invasion ability in A549 and H1299 cells with MRPL12 knockout or re-expression. **F** Quantification of A549 and H1299 cells in trans-endothelial migration assays (referenced in Figure 3H). **G-H** Ingenuity Pathway Analysis (IPA) of the differentially expressed genes in A549 cells with MRPL12 knockdown. *, p<0.05; **, p<0.01; ***, p<0.001; ****, p<0.0001.

**Figure S3. Co‐expression analysis combined with KEGG enrichment analysis**

**A** Co‐expression analysis of MRPL12. **B** Western blotting was used to analyze the expression of the indicated genes following MRPL12 knockdown or overexpression in A549 and H1299 cells. *, p<0.05; **, p<0.01; ***, p<0.001, ****, p<0.0001; ns, p>0.05.

**Figure S4. MRPL12 promotes lung tumorigenesis via promoting mitochondrial oxidative phosphorylation**

**A** Analysis of mtDNA copy number in H1299 cells subjected to MRPL12 knockdown or overexpression. **B-C** Evaluation of mRNA (B) and protein (C) levels of OXPHOS complexes in H1299 cells following MRPL12 knockdown or overexpression. **D** Measurement of OCR in H1299 cells with MRPL12 knockdown or overexpression using Seahorse XFe96. **E** Analysis of mitochondrial OXPHOS, including basal respiration, maximal respiration, ATP production, and spare respiratory capacity in H1299 cells with MRPL12 knockdown or overexpression. **F** Mitochondrial mass was measured using MitoTracker Red CMXRos. **G-H** Examination of the proliferation and migration capabilities of A549 and H1299 cells overexpressing MRPL12 under treatment with an OXPHOS inhibitor. *, p<0.05; **, p<0.01; ***, p<0.001; ****, p<0.0001; ns, p>0.05.

**Figure S5. UBASH3B interacts with MRPL12**

**A** Analysis of UBASH3B expression across various cancer types using pan-cancer samples from the TIMER2.0 database. **B** Examination of UBASH3B expression in LUAD and corresponding normal tissues within the TCGA databases. **C** Assessment of UBASH3B expression in LUAD and adjacent normal tissues through analysis of the TCGA databases. **D** Comparative analysis of UBASH3B expression in LUAD and normal tissues utilizing the GEPIA2 website. **E-F** Kaplan-Meier survival curves illustrating the impact of UBASH3B expression on the overall survival of LUAD patients. **G-H** Evaluation of the migration (**G**) and proliferation (**H**) capabilities of A549 and H1299 cells with UBASH3B overexpression or knockdown. **I-J** Investigation of the proliferation and migration properties of A549 and H1299 cells overexpressing MRPL12, along with UBASH3B H391A or UBASH3B WT overexpression, assessed by EDU and Transwell assays. *, p<0.05; **, p<0.01; ***, p<0.001; ****, p<0.0001.

**Figure S6. The expression and role of UBASH3B in LUAD**

**A** UBASH3B expression was assessed in LUAD tissues and PDO. **B-C** Analysis of the correlation between UBASH3B expression levels and TNM stages or pathological stage using TCGA datasets. **D** Transwell assays were conducted to assess cell migration and invasion ability in A549 and H1299 cells with UBASH3B knockdown, combined with MRPL12 WT, MRPL12 Y60A or MRPL12 Y60E overexpression. **E** EDU assays were conducted to assess cell proliferation in A549 and H1299 cells with UBASH3B overexpression, combined with MRPL12 WT, MRPL12 Y60E or MRPL12 Y60A overexpression. *, p<0.05; **, p<0.01; ***, p<0.001; ****, p<0.0001.

**Figure S7. UBASH3B dephosphorylates MRPL12 at residue Y60 and inhibits lung tumorigenesis**

**A-B** Assessment of protein (A) and mRNA (B) levels of OXPHOS complexes in H1299 cells overexpressing the MRPL12 Y60A mutation or MRPL12 WT. **C** Analysis of mtDNA copy number in H1299 cells with overexpression of MRPL12 WT or the MRPL12 Y60A mutant. **D** Examination of oxygen consumption rate (OCR) in H1299 cells overexpressing MRPL12 Y60A or MRPL12 WT, performed using Seahorse XFe96. **E** Quantification of basal respiration, ATP production, maximal respiration, and spare respiratory capacity in H1299 cells overexpressing MRPL12 Y60A or MRPL12 WT. **F** Co-ip assays conducted in A549 and H1299 cells, wherein cells were lysed and immunoprecipitated with an MRPL12 antibody, followed by western blot analysis with POLRMT. **G-H** Analysis of proliferation and migration characteristics in A549 and H1299 cells with overexpression of MRPL12 WT, MRPL12 Y60A, or Y152A mutations. *, p<0.05; **, p<0.01; ***, p<0.001; ****, p<0.0001; ns, p>0.05.
