# Supplementary figures and images for: UBASH3B-mediated MRPL12 Y60 dephosphorylation inhibits LUAD development by driving mitochondrial metabolism reprogramming
Source: J Exp Clin Cancer Res. 2024 Sep 30;43:268. doi: 10.1186/s13046-024-03181-x (PMC11441236; doi:10.1186/s13046-024-03181-x)

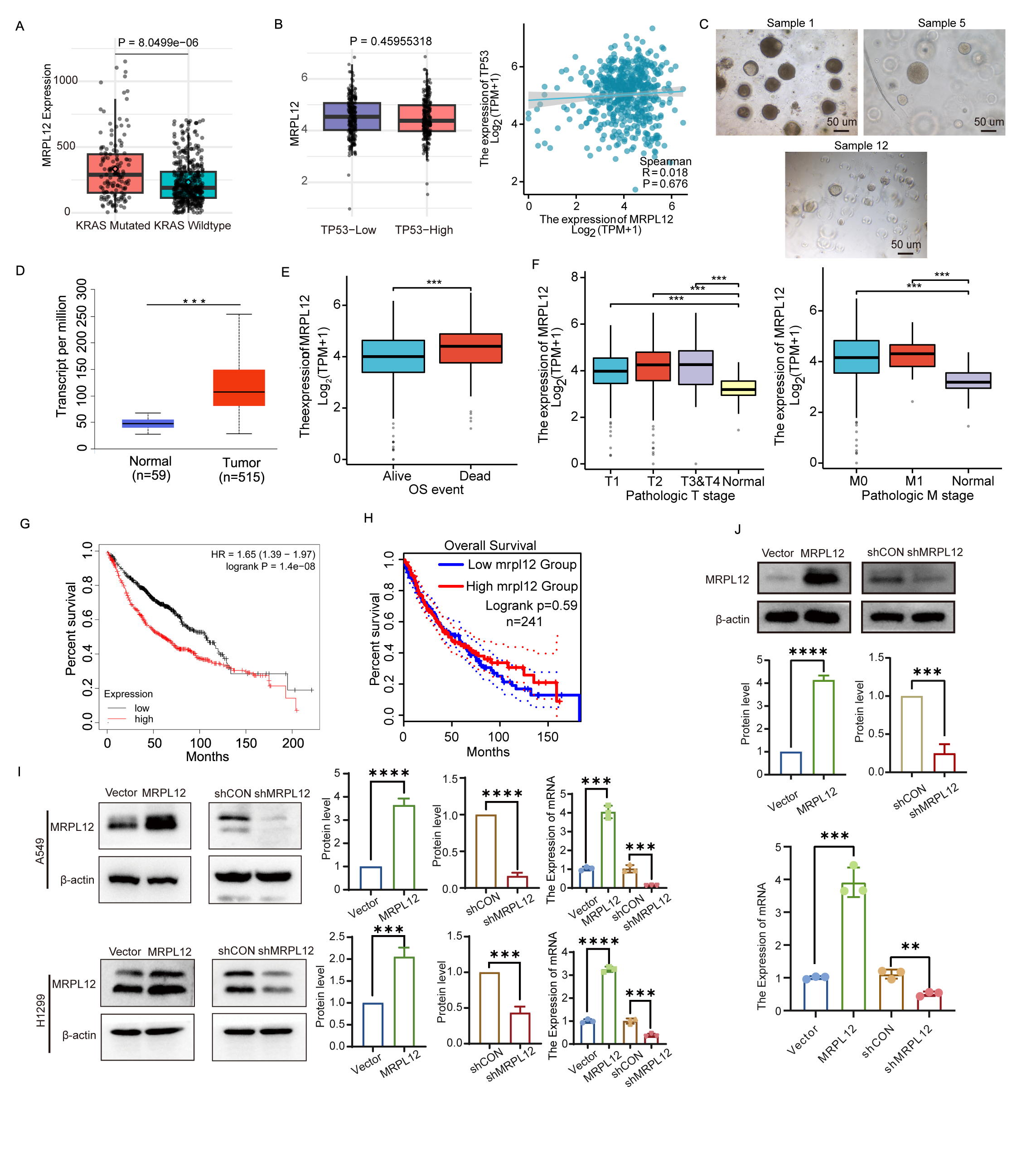

Supplement: Supplementary file 1 — Supplementary Material 1: Figure S1. MRPL12 is highly expressed in LUAD organoid, tissues, and cells and associated with poor survival. A Analysis of MRPL12 expression in KRAS mutant versus non-mutant LUAD tissues using TCGA databases. B Analysis of the correlation between MRPL12 and TP53 expression in LUAD tissues using TCGA databases. C Organoid morphology. Scale bar: 50 μm. D MRPL12 mRNA levels in LUAD. E MRPL12 mRNA levels in deceased and living LUAD patients. F Analysis of MRPL12 expression levels based on T, N, M clinical stage, and pathologic stage. G Overall survival analysis based on MRPL12 mRNA levels in LUAD. H Kaplan-Meier survival curves depicting the correlation between MRPL12 mRNA levels and the overall survival time of patients with lung squamous cell carcinoma. I-J Western blot and RT-PCR were employed to assess the efficiency of MRPL12 knockdown or overexpression in the indicated cells (I) and PDOs (J). **, p<0.01;***, p<0.001; ****, p<0.0001. Figure S2. MRPL12 facilitates LUAD tumorigenesis in vitro. A-B Soft agar (A) and EDU (B) assays were conducted to evaluate cell proliferation and colony formation capabilities in A549 and H1299 cells with stable MRPL12 overexpression or knockdown. C EDU assays were performed to assess cell proliferation in A549 and H1299 cells with MRPL12 knockout or re-expression. D Transwell assays were conducted to assess cell migration and invasion ability in A549 and H1299 cells with stable MRPL12 overexpression or knockdown. E Transwell assays were conducted to assess cell migration and invasion ability in A549 and H1299 cells with MRPL12 knockout or re-expression. F Quantification of A549 and H1299 cells in trans-endothelial migration assays (referenced in Figure 3H). G-H Ingenuity Pathway Analysis (IPA) of the differentially expressed genes in A549 cells with MRPL12 knockdown. *, p<0.05; **, p<0.01; ***, p<0.001; ****, p<0.0001. Figure S3. Co‐expression analysis combined with KEGG enrichment analysis. A Co‐expression [file 13046_2024_3181_MOESM1_ESM.zip › 13046_2024_3181_MOESM1_ESM/13046_2024_3181_MOESM1_ESM.tif]

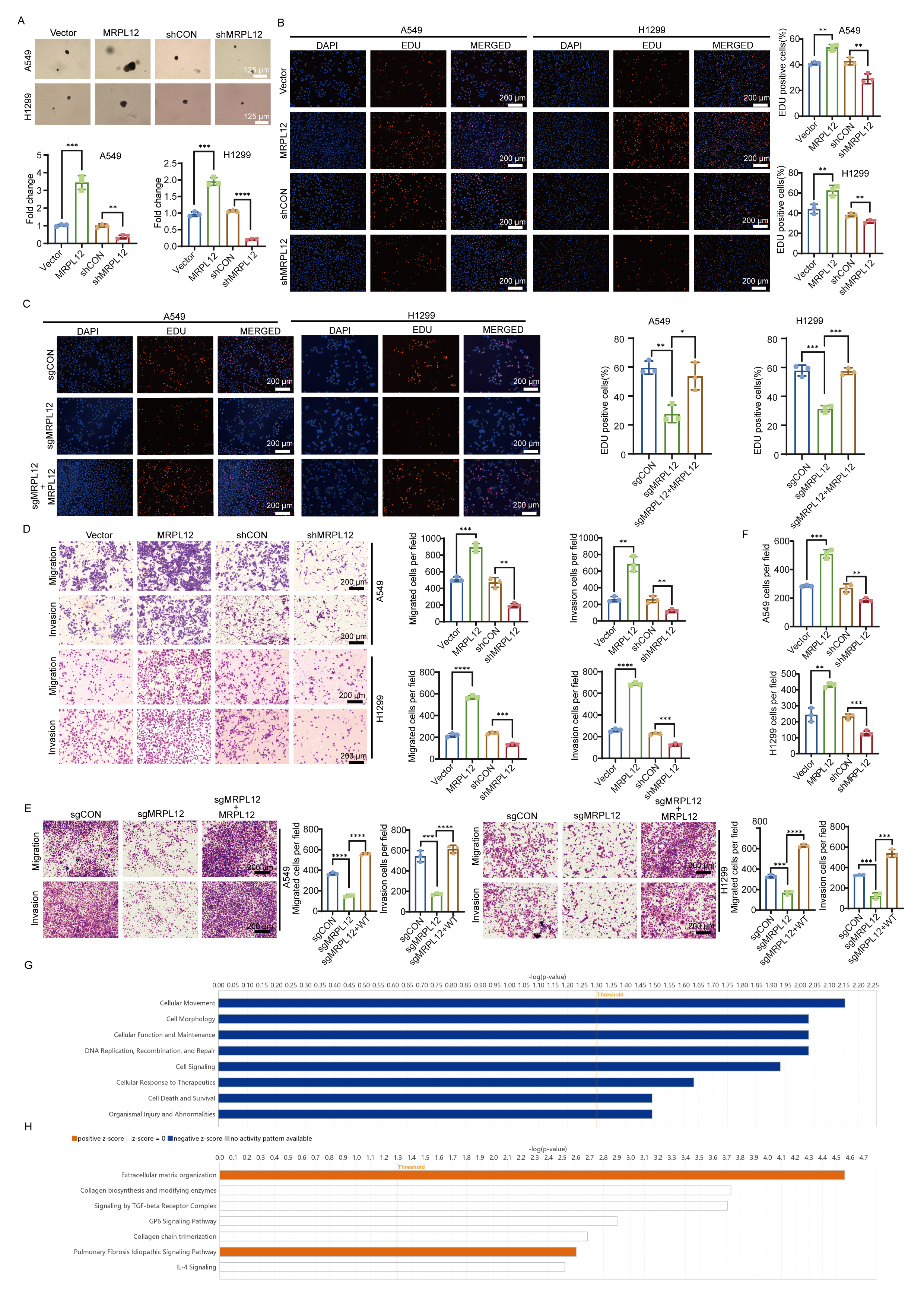

Supplement: Supplementary file 1 — Supplementary Material 1: Figure S1. MRPL12 is highly expressed in LUAD organoid, tissues, and cells and associated with poor survival. A Analysis of MRPL12 expression in KRAS mutant versus non-mutant LUAD tissues using TCGA databases. B Analysis of the correlation between MRPL12 and TP53 expression in LUAD tissues using TCGA databases. C Organoid morphology. Scale bar: 50 μm. D MRPL12 mRNA levels in LUAD. E MRPL12 mRNA levels in deceased and living LUAD patients. F Analysis of MRPL12 expression levels based on T, N, M clinical stage, and pathologic stage. G Overall survival analysis based on MRPL12 mRNA levels in LUAD. H Kaplan-Meier survival curves depicting the correlation between MRPL12 mRNA levels and the overall survival time of patients with lung squamous cell carcinoma. I-J Western blot and RT-PCR were employed to assess the efficiency of MRPL12 knockdown or overexpression in the indicated cells (I) and PDOs (J). **, p<0.01;***, p<0.001; ****, p<0.0001. Figure S2. MRPL12 facilitates LUAD tumorigenesis in vitro. A-B Soft agar (A) and EDU (B) assays were conducted to evaluate cell proliferation and colony formation capabilities in A549 and H1299 cells with stable MRPL12 overexpression or knockdown. C EDU assays were performed to assess cell proliferation in A549 and H1299 cells with MRPL12 knockout or re-expression. D Transwell assays were conducted to assess cell migration and invasion ability in A549 and H1299 cells with stable MRPL12 overexpression or knockdown. E Transwell assays were conducted to assess cell migration and invasion ability in A549 and H1299 cells with MRPL12 knockout or re-expression. F Quantification of A549 and H1299 cells in trans-endothelial migration assays (referenced in Figure 3H). G-H Ingenuity Pathway Analysis (IPA) of the differentially expressed genes in A549 cells with MRPL12 knockdown. *, p<0.05; **, p<0.01; ***, p<0.001; ****, p<0.0001. Figure S3. Co‐expression analysis combined with KEGG enrichment analysis. A Co‐expression [file 13046_2024_3181_MOESM1_ESM.zip › 13046_2024_3181_MOESM1_ESM/13046_2024_3181_MOESM2_ESM.tif]

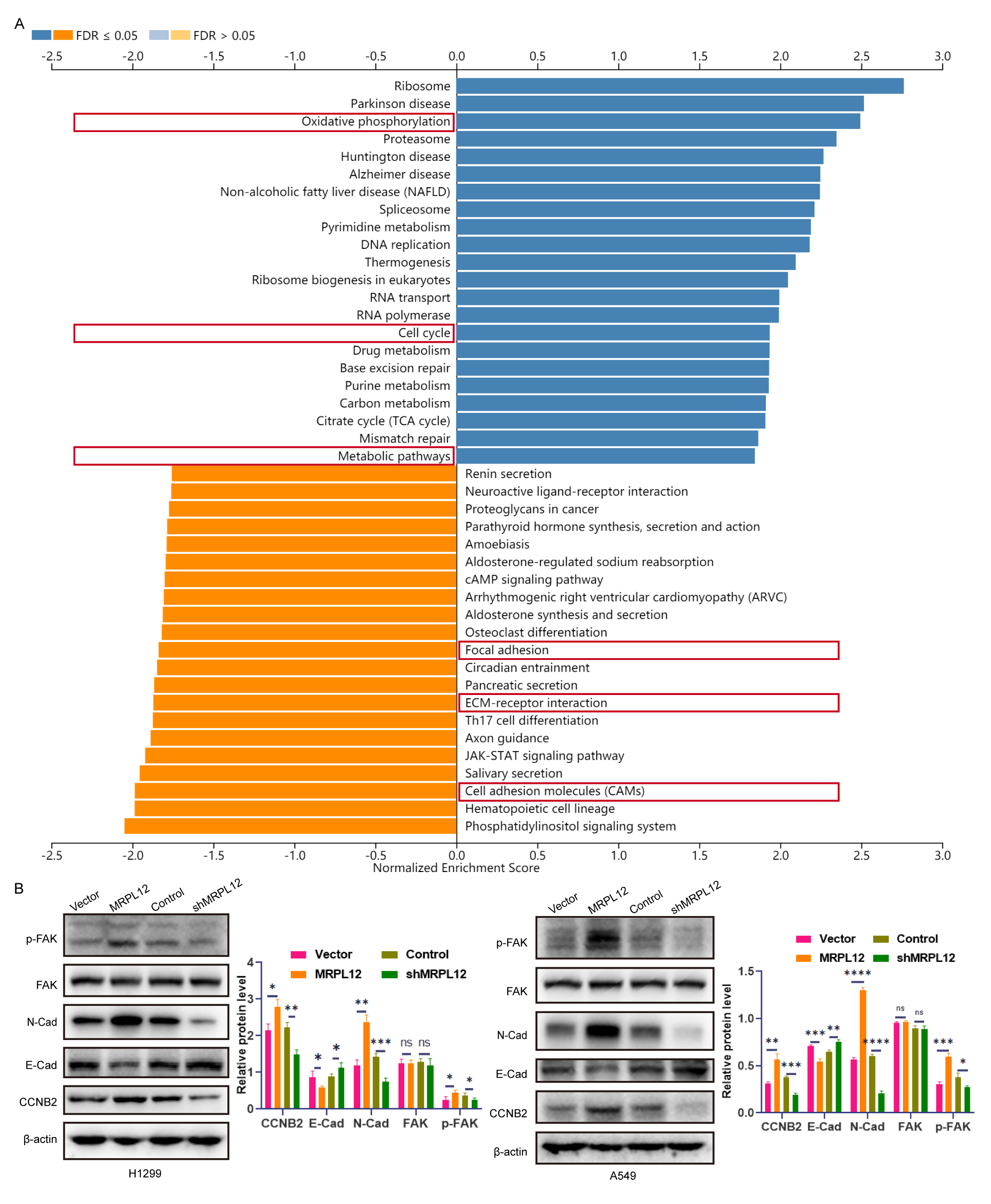

Supplement: Supplementary file 1 — Supplementary Material 1: Figure S1. MRPL12 is highly expressed in LUAD organoid, tissues, and cells and associated with poor survival. A Analysis of MRPL12 expression in KRAS mutant versus non-mutant LUAD tissues using TCGA databases. B Analysis of the correlation between MRPL12 and TP53 expression in LUAD tissues using TCGA databases. C Organoid morphology. Scale bar: 50 μm. D MRPL12 mRNA levels in LUAD. E MRPL12 mRNA levels in deceased and living LUAD patients. F Analysis of MRPL12 expression levels based on T, N, M clinical stage, and pathologic stage. G Overall survival analysis based on MRPL12 mRNA levels in LUAD. H Kaplan-Meier survival curves depicting the correlation between MRPL12 mRNA levels and the overall survival time of patients with lung squamous cell carcinoma. I-J Western blot and RT-PCR were employed to assess the efficiency of MRPL12 knockdown or overexpression in the indicated cells (I) and PDOs (J). **, p<0.01;***, p<0.001; ****, p<0.0001. Figure S2. MRPL12 facilitates LUAD tumorigenesis in vitro. A-B Soft agar (A) and EDU (B) assays were conducted to evaluate cell proliferation and colony formation capabilities in A549 and H1299 cells with stable MRPL12 overexpression or knockdown. C EDU assays were performed to assess cell proliferation in A549 and H1299 cells with MRPL12 knockout or re-expression. D Transwell assays were conducted to assess cell migration and invasion ability in A549 and H1299 cells with stable MRPL12 overexpression or knockdown. E Transwell assays were conducted to assess cell migration and invasion ability in A549 and H1299 cells with MRPL12 knockout or re-expression. F Quantification of A549 and H1299 cells in trans-endothelial migration assays (referenced in Figure 3H). G-H Ingenuity Pathway Analysis (IPA) of the differentially expressed genes in A549 cells with MRPL12 knockdown. *, p<0.05; **, p<0.01; ***, p<0.001; ****, p<0.0001. Figure S3. Co‐expression analysis combined with KEGG enrichment analysis. A Co‐expression [file 13046_2024_3181_MOESM1_ESM.zip › 13046_2024_3181_MOESM1_ESM/13046_2024_3181_MOESM3_ESM.tif]

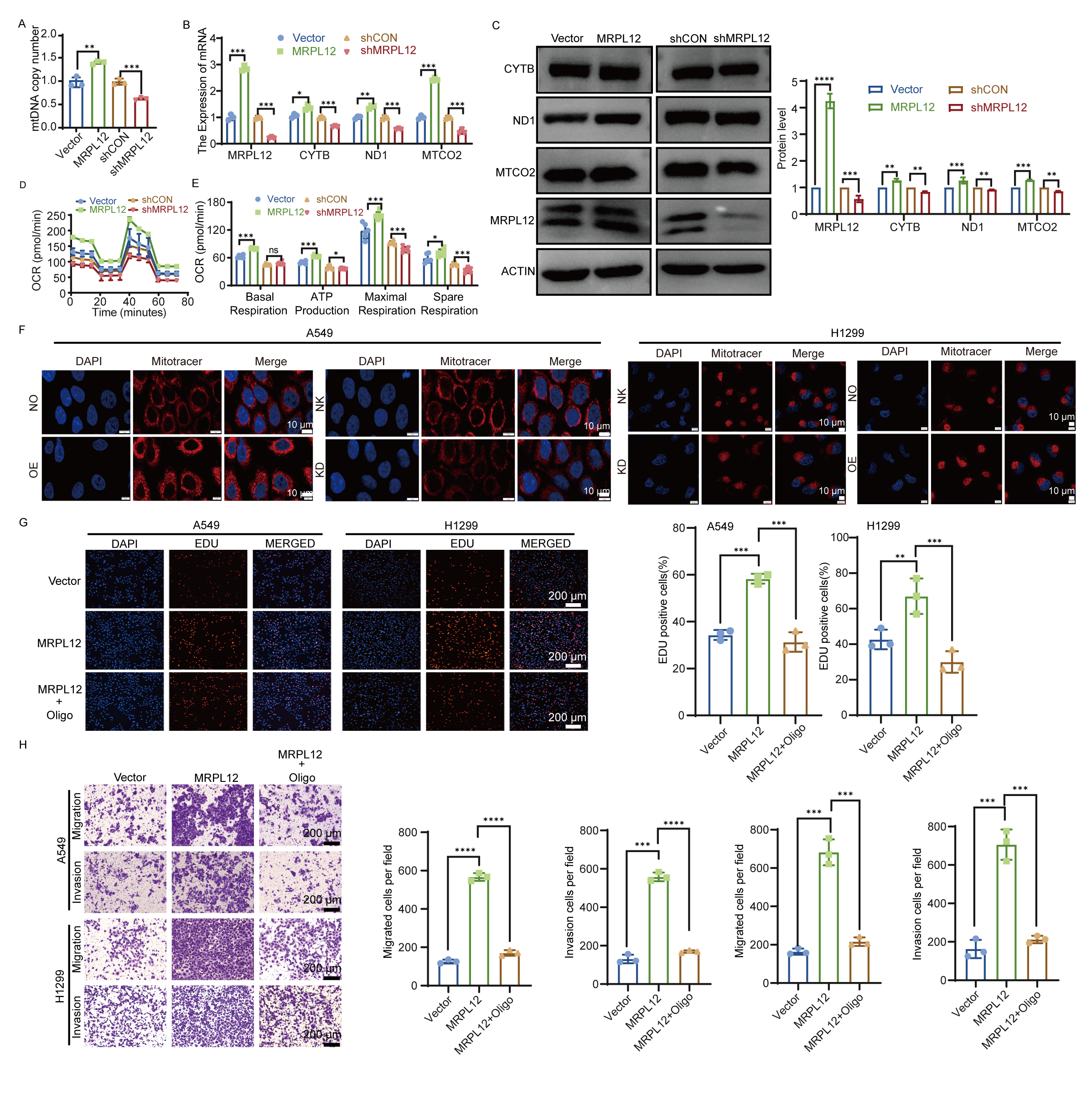

Supplement: Supplementary file 1 — Supplementary Material 1: Figure S1. MRPL12 is highly expressed in LUAD organoid, tissues, and cells and associated with poor survival. A Analysis of MRPL12 expression in KRAS mutant versus non-mutant LUAD tissues using TCGA databases. B Analysis of the correlation between MRPL12 and TP53 expression in LUAD tissues using TCGA databases. C Organoid morphology. Scale bar: 50 μm. D MRPL12 mRNA levels in LUAD. E MRPL12 mRNA levels in deceased and living LUAD patients. F Analysis of MRPL12 expression levels based on T, N, M clinical stage, and pathologic stage. G Overall survival analysis based on MRPL12 mRNA levels in LUAD. H Kaplan-Meier survival curves depicting the correlation between MRPL12 mRNA levels and the overall survival time of patients with lung squamous cell carcinoma. I-J Western blot and RT-PCR were employed to assess the efficiency of MRPL12 knockdown or overexpression in the indicated cells (I) and PDOs (J). **, p<0.01;***, p<0.001; ****, p<0.0001. Figure S2. MRPL12 facilitates LUAD tumorigenesis in vitro. A-B Soft agar (A) and EDU (B) assays were conducted to evaluate cell proliferation and colony formation capabilities in A549 and H1299 cells with stable MRPL12 overexpression or knockdown. C EDU assays were performed to assess cell proliferation in A549 and H1299 cells with MRPL12 knockout or re-expression. D Transwell assays were conducted to assess cell migration and invasion ability in A549 and H1299 cells with stable MRPL12 overexpression or knockdown. E Transwell assays were conducted to assess cell migration and invasion ability in A549 and H1299 cells with MRPL12 knockout or re-expression. F Quantification of A549 and H1299 cells in trans-endothelial migration assays (referenced in Figure 3H). G-H Ingenuity Pathway Analysis (IPA) of the differentially expressed genes in A549 cells with MRPL12 knockdown. *, p<0.05; **, p<0.01; ***, p<0.001; ****, p<0.0001. Figure S3. Co‐expression analysis combined with KEGG enrichment analysis. A Co‐expression [file 13046_2024_3181_MOESM1_ESM.zip › 13046_2024_3181_MOESM1_ESM/13046_2024_3181_MOESM4_ESM.tif]

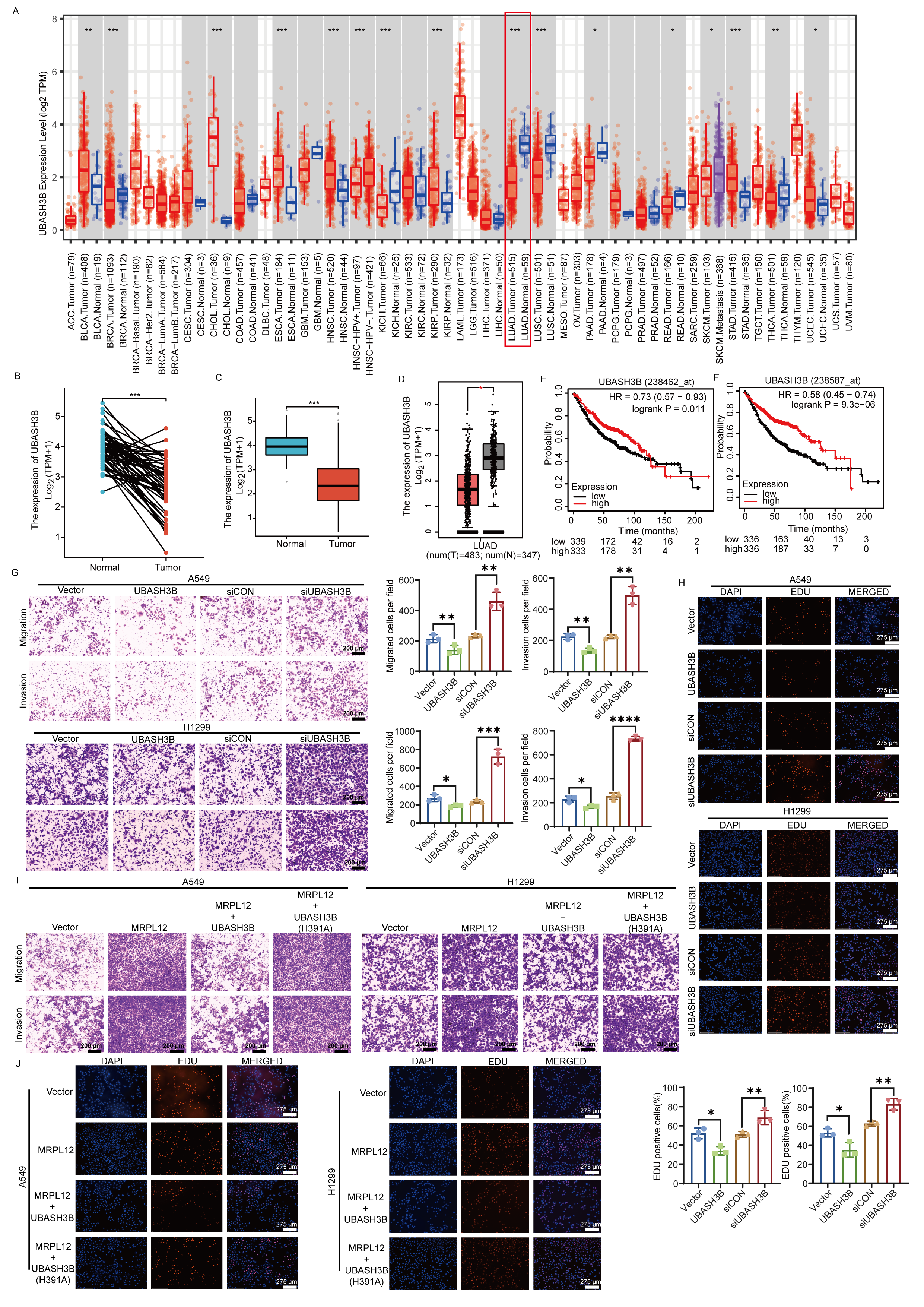

Supplement: Supplementary file 1 — Supplementary Material 1: Figure S1. MRPL12 is highly expressed in LUAD organoid, tissues, and cells and associated with poor survival. A Analysis of MRPL12 expression in KRAS mutant versus non-mutant LUAD tissues using TCGA databases. B Analysis of the correlation between MRPL12 and TP53 expression in LUAD tissues using TCGA databases. C Organoid morphology. Scale bar: 50 μm. D MRPL12 mRNA levels in LUAD. E MRPL12 mRNA levels in deceased and living LUAD patients. F Analysis of MRPL12 expression levels based on T, N, M clinical stage, and pathologic stage. G Overall survival analysis based on MRPL12 mRNA levels in LUAD. H Kaplan-Meier survival curves depicting the correlation between MRPL12 mRNA levels and the overall survival time of patients with lung squamous cell carcinoma. I-J Western blot and RT-PCR were employed to assess the efficiency of MRPL12 knockdown or overexpression in the indicated cells (I) and PDOs (J). **, p<0.01;***, p<0.001; ****, p<0.0001. Figure S2. MRPL12 facilitates LUAD tumorigenesis in vitro. A-B Soft agar (A) and EDU (B) assays were conducted to evaluate cell proliferation and colony formation capabilities in A549 and H1299 cells with stable MRPL12 overexpression or knockdown. C EDU assays were performed to assess cell proliferation in A549 and H1299 cells with MRPL12 knockout or re-expression. D Transwell assays were conducted to assess cell migration and invasion ability in A549 and H1299 cells with stable MRPL12 overexpression or knockdown. E Transwell assays were conducted to assess cell migration and invasion ability in A549 and H1299 cells with MRPL12 knockout or re-expression. F Quantification of A549 and H1299 cells in trans-endothelial migration assays (referenced in Figure 3H). G-H Ingenuity Pathway Analysis (IPA) of the differentially expressed genes in A549 cells with MRPL12 knockdown. *, p<0.05; **, p<0.01; ***, p<0.001; ****, p<0.0001. Figure S3. Co‐expression analysis combined with KEGG enrichment analysis. A Co‐expression [file 13046_2024_3181_MOESM1_ESM.zip › 13046_2024_3181_MOESM1_ESM/13046_2024_3181_MOESM5_ESM.tif]

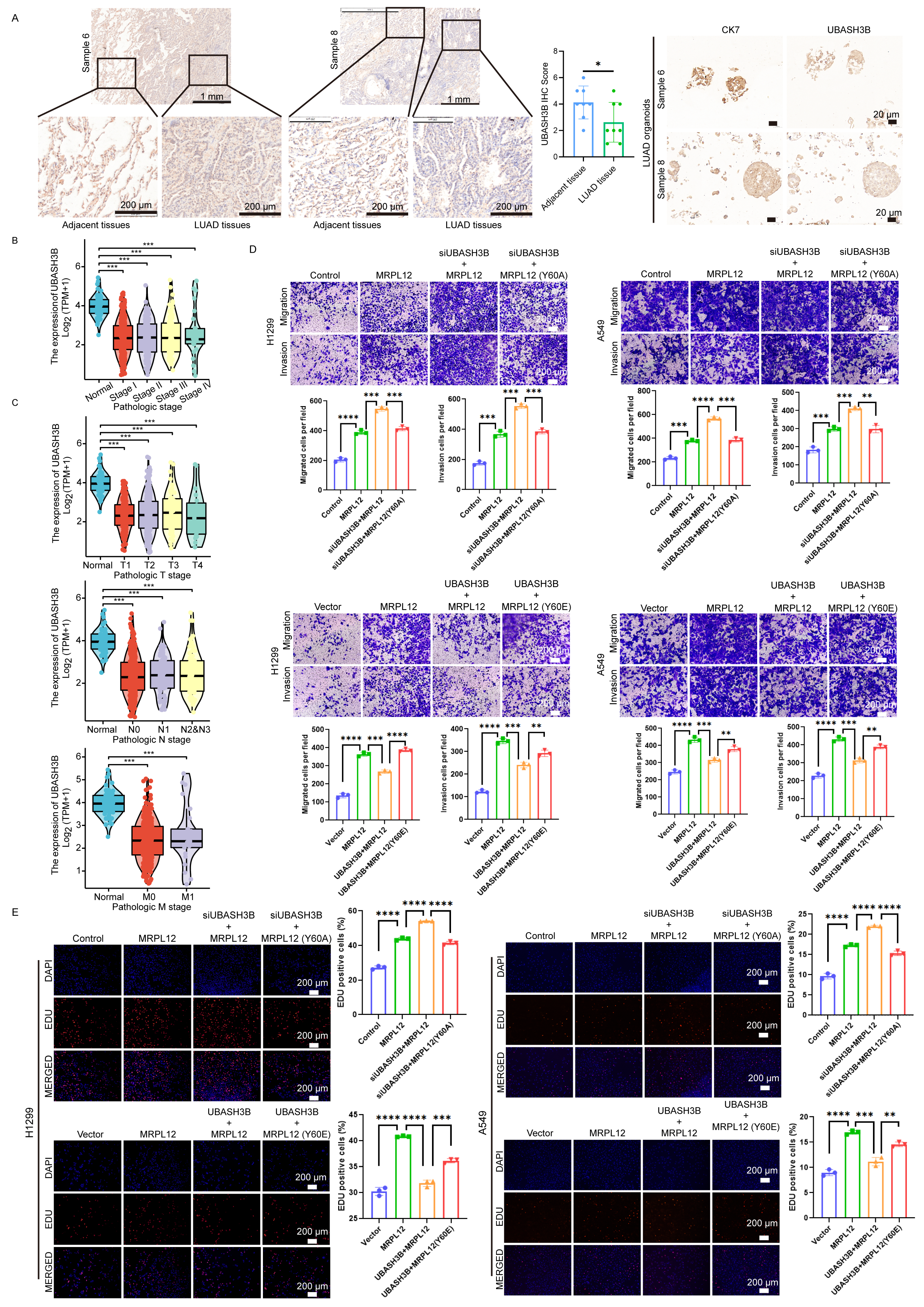

Supplement: Supplementary file 1 — Supplementary Material 1: Figure S1. MRPL12 is highly expressed in LUAD organoid, tissues, and cells and associated with poor survival. A Analysis of MRPL12 expression in KRAS mutant versus non-mutant LUAD tissues using TCGA databases. B Analysis of the correlation between MRPL12 and TP53 expression in LUAD tissues using TCGA databases. C Organoid morphology. Scale bar: 50 μm. D MRPL12 mRNA levels in LUAD. E MRPL12 mRNA levels in deceased and living LUAD patients. F Analysis of MRPL12 expression levels based on T, N, M clinical stage, and pathologic stage. G Overall survival analysis based on MRPL12 mRNA levels in LUAD. H Kaplan-Meier survival curves depicting the correlation between MRPL12 mRNA levels and the overall survival time of patients with lung squamous cell carcinoma. I-J Western blot and RT-PCR were employed to assess the efficiency of MRPL12 knockdown or overexpression in the indicated cells (I) and PDOs (J). **, p<0.01;***, p<0.001; ****, p<0.0001. Figure S2. MRPL12 facilitates LUAD tumorigenesis in vitro. A-B Soft agar (A) and EDU (B) assays were conducted to evaluate cell proliferation and colony formation capabilities in A549 and H1299 cells with stable MRPL12 overexpression or knockdown. C EDU assays were performed to assess cell proliferation in A549 and H1299 cells with MRPL12 knockout or re-expression. D Transwell assays were conducted to assess cell migration and invasion ability in A549 and H1299 cells with stable MRPL12 overexpression or knockdown. E Transwell assays were conducted to assess cell migration and invasion ability in A549 and H1299 cells with MRPL12 knockout or re-expression. F Quantification of A549 and H1299 cells in trans-endothelial migration assays (referenced in Figure 3H). G-H Ingenuity Pathway Analysis (IPA) of the differentially expressed genes in A549 cells with MRPL12 knockdown. *, p<0.05; **, p<0.01; ***, p<0.001; ****, p<0.0001. Figure S3. Co‐expression analysis combined with KEGG enrichment analysis. A Co‐expression [file 13046_2024_3181_MOESM1_ESM.zip › 13046_2024_3181_MOESM1_ESM/13046_2024_3181_MOESM6_ESM.tif]

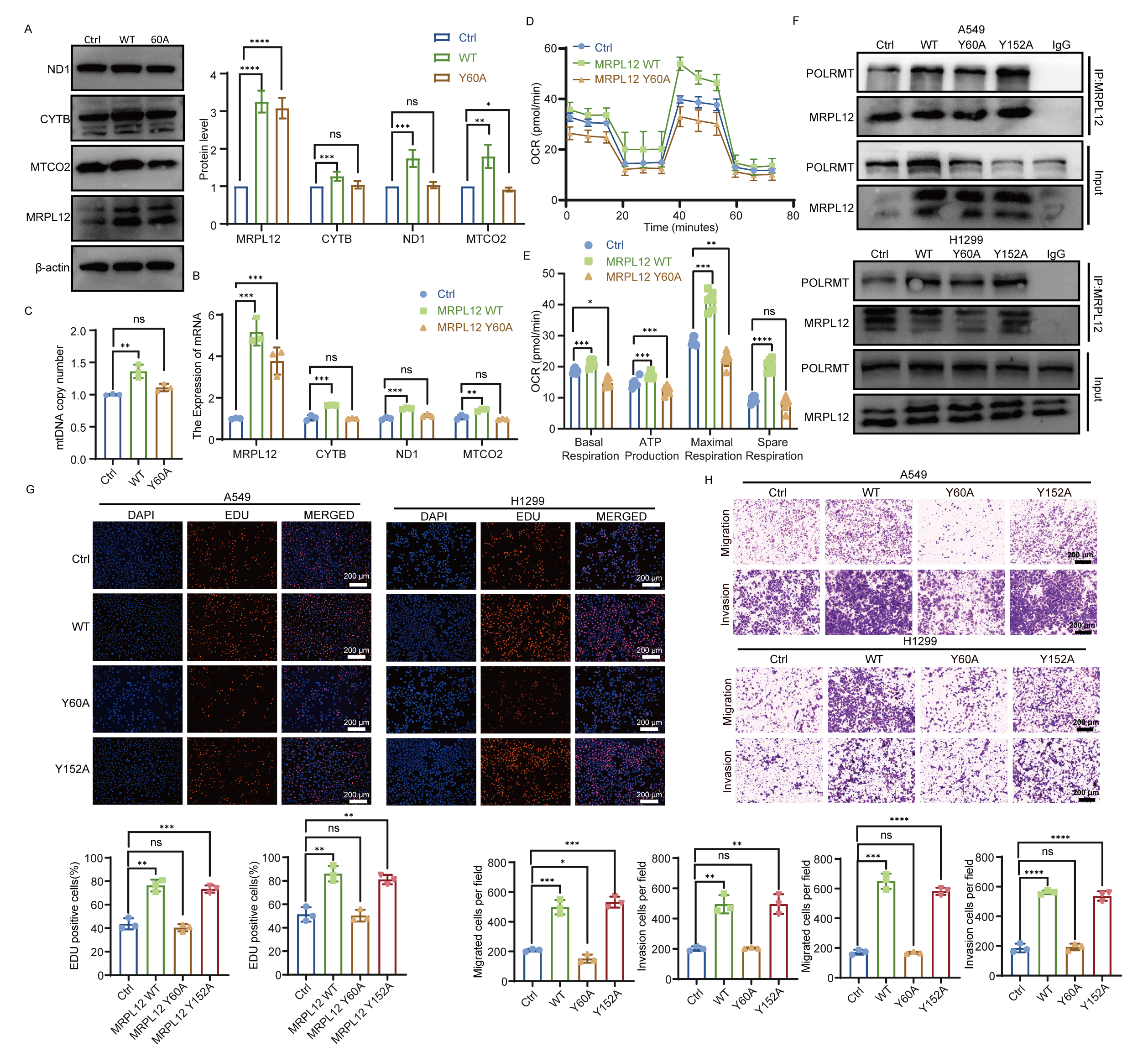

Supplement: Supplementary file 1 — Supplementary Material 1: Figure S1. MRPL12 is highly expressed in LUAD organoid, tissues, and cells and associated with poor survival. A Analysis of MRPL12 expression in KRAS mutant versus non-mutant LUAD tissues using TCGA databases. B Analysis of the correlation between MRPL12 and TP53 expression in LUAD tissues using TCGA databases. C Organoid morphology. Scale bar: 50 μm. D MRPL12 mRNA levels in LUAD. E MRPL12 mRNA levels in deceased and living LUAD patients. F Analysis of MRPL12 expression levels based on T, N, M clinical stage, and pathologic stage. G Overall survival analysis based on MRPL12 mRNA levels in LUAD. H Kaplan-Meier survival curves depicting the correlation between MRPL12 mRNA levels and the overall survival time of patients with lung squamous cell carcinoma. I-J Western blot and RT-PCR were employed to assess the efficiency of MRPL12 knockdown or overexpression in the indicated cells (I) and PDOs (J). **, p<0.01;***, p<0.001; ****, p<0.0001. Figure S2. MRPL12 facilitates LUAD tumorigenesis in vitro. A-B Soft agar (A) and EDU (B) assays were conducted to evaluate cell proliferation and colony formation capabilities in A549 and H1299 cells with stable MRPL12 overexpression or knockdown. C EDU assays were performed to assess cell proliferation in A549 and H1299 cells with MRPL12 knockout or re-expression. D Transwell assays were conducted to assess cell migration and invasion ability in A549 and H1299 cells with stable MRPL12 overexpression or knockdown. E Transwell assays were conducted to assess cell migration and invasion ability in A549 and H1299 cells with MRPL12 knockout or re-expression. F Quantification of A549 and H1299 cells in trans-endothelial migration assays (referenced in Figure 3H). G-H Ingenuity Pathway Analysis (IPA) of the differentially expressed genes in A549 cells with MRPL12 knockdown. *, p<0.05; **, p<0.01; ***, p<0.001; ****, p<0.0001. Figure S3. Co‐expression analysis combined with KEGG enrichment analysis. A Co‐expression [file 13046_2024_3181_MOESM1_ESM.zip › 13046_2024_3181_MOESM1_ESM/13046_2024_3181_MOESM7_ESM.tif]
